# An Isolated Complex V Inefficiency and Dysregulated Mitochondrial Function in Immortalized Lymphocytes from ME/CFS Patients

**DOI:** 10.3390/ijms21031074

**Published:** 2020-02-06

**Authors:** Daniel Missailidis, Sarah J. Annesley, Claire Y. Allan, Oana Sanislav, Brett A. Lidbury, Donald P. Lewis, Paul R. Fisher

**Affiliations:** 1 Department of Physiology, Anatomy, and Microbiology, La Trobe University, Melbourne, VIC 3086, Australia; dmissailidis@students.latrobe.edu.au (D.M.); S.Annesley@latrobe.edu.au (S.J.A.); Claire.Allan@latrobe.edu.au (C.Y.A.); O.Sanislav@latrobe.edu.au (O.S.); 2 National Centre for Epidemiology and Population Health, Research School of Population Health, Australian National University, Canberra, ACT 2601, Australia; brett.lidbury@anu.edu.au

**Keywords:** myalgic encephalomyelitis, chronic fatigue syndrome, mitochondria, Complex V, TORC1, seahorse respirometry

## Abstract

Myalgic encephalomyelitis/chronic fatigue syndrome (ME/CFS) is an enigmatic condition characterized by exacerbation of symptoms after exertion (post-exertional malaise or “PEM”), and by fatigue whose severity and associated requirement for rest are excessive and disproportionate to the fatigue-inducing activity. There is no definitive molecular marker or known underlying pathological mechanism for the condition. Increasing evidence for aberrant energy metabolism suggests a role for mitochondrial dysfunction in ME/CFS. Our objective was therefore to measure mitochondrial function and cellular stress sensing in actively metabolizing patient blood cells. We immortalized lymphoblasts isolated from 51 ME/CFS patients diagnosed according to the Canadian Consensus Criteria and an age- and gender-matched control group. Parameters of mitochondrial function and energy stress sensing were assessed by Seahorse extracellular flux analysis, proteomics, and an array of additional biochemical assays. As a proportion of the basal oxygen consumption rate (OCR), the rate of ATP synthesis by Complex V was significantly reduced in ME/CFS lymphoblasts, while significant elevations were observed in Complex I OCR, maximum OCR, spare respiratory capacity, nonmitochondrial OCR and “proton leak” as a proportion of the basal OCR. This was accompanied by a reduction of mitochondrial membrane potential, chronically hyperactivated TOR Complex I stress signaling and upregulated expression of mitochondrial respiratory complexes, fatty acid transporters, and enzymes of the β-oxidation and TCA cycles. By contrast, mitochondrial mass and genome copy number, as well as glycolytic rates and steady state ATP levels were unchanged. Our results suggest a model in which ME/CFS lymphoblasts have a Complex V defect accompanied by compensatory upregulation of their respiratory capacity that includes the mitochondrial respiratory complexes, membrane transporters and enzymes involved in fatty acid β-oxidation. This homeostatically returns ATP synthesis and steady state levels to “normal” in the resting cells, but may leave them unable to adequately respond to acute increases in energy demand as the relevant homeostatic pathways are already activated.

## 1. Introduction

Myalgic encephalomyelitis (ME), also referred to as chronic fatigue syndrome (CFS), is a condition which is little understood, its hallmarks being chronic, unexplained fatigue and the debilitating overexertion “payback” termed post exertional malaise (PEM) experienced by patients [1]. PEM can occur after even the simplest of daily physical tasks and may be accompanied by symptoms affecting a range of body systems. The lack of objective, timely, and accurate diagnostic criteria leaves patients for long periods without a clear diagnosis or an informed understanding of the condition [1,2]. It is paramount that fundamental molecular explanations for the underlying pathophysiology of ME/CFS and reliable biomarkers are pursued. These could lead to reliable, faster diagnosis and, in the longer term, rational, effective treatments.

Mitochondrial function is of interest to ME/CFS research as a bioenergetic explanation for the recurrent fatigue and the myriad links between the disorder’s characteristic immune inflammation and the mitochondria [3]. Evidence for mitochondrial dysfunction in ME/CFS has been sought in the last decade, yet remains inconsistent. Reduced mitochondrial biogenesis but not normalized respiratory chain enzyme activities have been reported in the muscle of ME/CFS individuals [4]. Muscle mitochondrial biogenesis is upregulated by exercise [5], so this reduction is likely to be caused by the necessarily reduced exercise that ME/CFS patients can undertake. Mitochondrial respiratory function in ME/CFS neutrophils [6,7] and peripheral blood mononuclear cells (PBMCs) is reportedly reduced [8] or unchanged [9], yet the oxidative phosphorylation (OXPHOS) complexes appear normal [10,11], while the expression of genes encoding mitochondrial proteins in patient saliva, platelets, and lymphocytes is elevated [12,13,14]. Most recently, mitochondrial respiration was found to be unchanged in resting and stimulated CD4^+^ and CD8^+^ T cells, with the sole exceptions of a small reduction in the proton leak in resting and ATP synthesis in stimulated CD8^+^ cells [15].

Differences between patient and control serum and urine metabolomes have been attributed to reduced provision of acetyl CoA to the TCA cycle caused by a defect in glycolysis [16] or by a defect in pyruvate dehydrogenase (PDH) [17]. While inconsistent, both proposals draw attention to a potential role in ME/CFS for impaired provision of reducing equivalents to mitochondrial OXPHOS by the TCA cycle. Despite their shared conviction that ME/CFS cells have fundamental problems in energy metabolism, the conflicting reports on the nature of these problems prompted us to reexamine the issue of mitochondrial function and its regulation in ME/CFS cells.

A key regulator of mitochondrial function is TORC1 (Target of Rapamycin Complex I) which regulates cell growth and energetics in a variety of cellular stress-sensing pathways [18]. This pathway upregulates the expression of nuclear-encoded mitochondrial proteins [19], among which are subunits of the OXPHOS complexes [20]. Despite this connection, and the agency of TORC1 within a complex regulatory network which responds to intracellular stressors including energy supply, dysregulation of this signaling pathway has not yet been investigated in ME/CFS cells.

To clarify the roles of aberrant mitochondrial function and TORC1 signaling in ME/CFS, we have compared parameters of mitochondrial function in immortalized lymphocytes (termed lymphoblasts) from patient blood with those from healthy age- and gender-matched controls. We found that mitochondrial function in ME/CFS lymphoblasts is indeed abnormal, with an isolated Complex V deficiency accompanied by elevated capacity of Complexes I to IV, decreased membrane potential, upregulation of TORC1 activity and elevated expression of diverse mitochondrial proteins involved ATP-generating catabolic pathways. This pattern of changes in mitochondrial function in ME/CFS lymphoblasts is distinct from what we observed using the same approach to other neurological conditions, for example the mitochondrial hyperactivity we reported previously in Parkinson’s disease lymphoblasts [21] and fragile X-associated tremor/ataxia syndrome lymphoblasts [22]. It suggests a model involving a primary deficiency in Complex V function, combined with homeostatic, compensatory upregulation of TORC1 activity and mitochondrial protein expression.

## 2. Results

### 2.1. Ex Vivo Lymphocytes Are Metabolically Quiescent and Those from ME/CFS Patients Die More Rapidly than Controls

**Figure 1:**
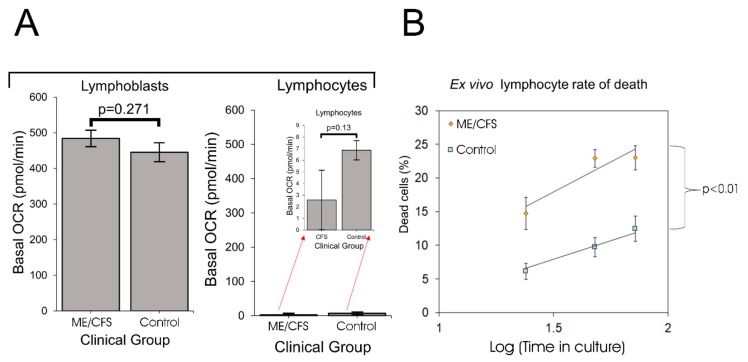
Ex vivo lymphocytes are metabolically quiescent and myalgic encephalomyelitis/chronic fatigue syndrome (ME/CFS) lymphocytes die more rapidly. Error bars are standard errors of the mean. (**A**) Basal oxygen consumption rates (OCR) was measured in lymphoblasts and lymphocytes from ME/CFS and control individuals. Lymphoblasts: each ME/CFS (*n* = 50) and control (*n* = 22) cell line was assayed over four replicates in at least three independent experiments. Lymphocytes: each ME/CFS (*n* = 14) and control (*n* = 9) cell line was assayed over four replicates once due to limited supply. The red arrows point to the same data magnified with a smaller Y axis scale. The low basal OCRs for lymphocytes match those previously reported [8]. (**B**) ME/CFS lymphocytes die more rapidly than healthy controls. Lymphocytes from ME/CFS patients (*n* = 35) and healthy controls (*n* = 14) were seeded at a density of 1 × 10^6^ viable cells/mL in RPMI 1640 with 10% serum and kept in a humidified 5% CO_2_ incubator at 37 °C during the experiment. Each point represents the mean percentage of dead cells at the corresponding time point for ex vivo lymphocytes from ME/CFS patients and healthy controls. Stepwise multiple regression analysis was performed with dummy variables allowing both slopes and intercepts to differ between groups, with removal of least significant regression variables until only significant coefficients remained. The difference in the slopes (death rates) of the log-linear regressions between the ME/CFS and control group was statistically significant (*t* test).

Whereas mitochondrial protein expression is elevated in ME/CFS saliva, lymphocytes and platelets [12,13,23], physiological measures of respiratory function and capacity in ME/CFS lymphocyte mitochondria are reportedly reduced [6,8]. A possible explanation is that the ex vivo ME/CFS lymphocytes are more deeply quiescent (metabolism more suppressed) than control cells. We investigated this by comparing respiration rates in immortalized lymphocytes (lymphoblasts) from ME/CFS patients and controls with those of lymphocytes from a random subset (determined by lymphocyte supply) of the same participant cohort. Although the ME/CFS lymphocytes appeared to have smaller respiration rates than controls, the difference was not significant. The basal respiration rates in both patient and control lymphocytes were two orders of magnitude lower than in the immortalized cells, approaching the lower limits of detectability in the instrument (Figure 1A, Figure S1), as in previously reported experiments with this cell type [8]. This confirmed that ex vivo lymphocytes from both patients and controls are in a deep state of physiological quiescence and so perhaps not representative of metabolically active cells in vivo.

Another potential contributor to the reported reduction in mitochondrial activity in ME/CFS lymphocytes compared to controls, is an increased death rate in ME/CFS lymphocytes compared to controls. We therefore assessed the viability over time of ME/CFS lymphocytes versus healthy controls (Figure 1B). In multiple log-linear regression analysis, the intercepts (which in the log-linear regression corresponds to an incubation time of 1 h) and the difference between them were not statistically significant. Although an extrapolation, this suggests that in both ME/CFS and control samples the fraction of dead cells at the start of the incubation was small and was similar in the two groups. However, the death rate was significant in both ME/CFS and control samples and was dramatically higher in the ME/CFS lymphocytes than in the controls. This suggests that previously reported reductions in ME/CFS lymphocyte mitochondrial function might have resulted from a higher fraction of dead cells in the assayed population. If it reflects the in vivo life span of unactivated lymphocytes, this result would also suggest that the turnover of unactivated lymphocytes in ME/CFS patients may be elevated.

### 2.2. ATP Synthesis by Complex V Is Inefficient in ME/CFS Lymphoblasts

The foregoing results suggest that lymphoblastoid cell lines (lymphoblasts) may better reflect the function of actively metabolizing cells in vivo, including activated leukocytes such as may be involved in inflammatory processes in ME/CFS patients. We therefore used lymphoblasts in the remainder of this study to investigate mitochondrial function in ME/CFS cells. Creation of the lymphoblasts involves immortalization by EBV infection and integration of the EBV genome into the lymphocyte genome. To check for possible effects of EBV on differences in mitochondrial and cellular stress signaling parameters between patient and control groups, we assayed EBV genome copy numbers (by qPCR) and found no significant difference between ME/CFS and control lymphoblasts (Figure S4). Furthermore, there was no effect of EBV genome copy number in either the patient or the control group on any of the mitochondrial and cell stress-signaling parameters we measured (Pearson, Spearman rank, and Kendall’s tau correlation coefficients, *p* > 0.05 in all cases).

**Figure 2:**
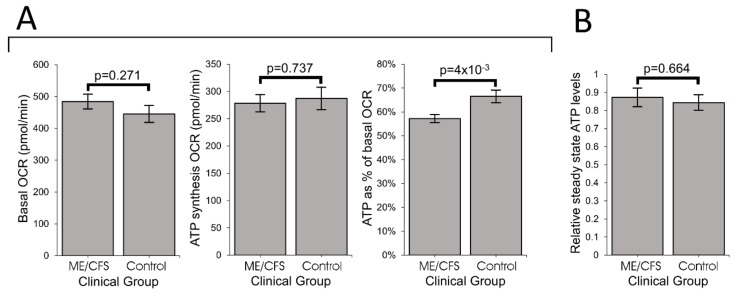
ATP synthesis by Complex V is inefficient in ME/CFS lymphoblasts. Error bars are standard errors of the mean. (**A**) Basal OCR and OCR by ATP synthesis were unchanged while OCR by ATP synthesis as a% of basal OCR was reduced in ME/CFS lymphoblasts (independent *t*-test). Each ME/CFS (*n* = 50) and control (*n* = 22) cell line was assayed over four replicates in at least three independent experiments. (**B**) Intracellular ATP concentration (relative background-subtracted luciferase luminescence) is unchanged in ME/CFS lymphoblasts (independent *t*-test). Each ME/CFS (*n* = 49) and control (*n* = 22) cell line was assayed in duplicate within each of at least three independent experiments. Data is normalized to an internal control cell line.

In ME/CFS lymphoblasts, basal respiration was slightly elevated and the rate of O_2_ consumption by ATP synthesis (oligomycin-sensitive component of basal respiration) slightly depressed, but neither change was statistically significant. However, as a proportion of the basal OCR, the rate of ATP synthesis by Complex V was significantly reduced (by about 15% relative to controls) in ME/CFS lymphoblasts, indicating an inefficiency in respiratory ATP synthesis (Figure 2A, Figure S5 Panel 1A).

Since the absolute rate of ATP synthesis was not significantly altered, despite its inefficiency, we anticipated that resting ME/CFS cells homeostatically maintain normal ATP levels. To verify this, we assayed whole cell ATP levels and found no difference between ME/CFS and control lymphoblasts (Figure 2B, Figure S5 Panel 1B).

### 2.3. ME/CFS Lymphoblasts Exhibit Elevated Respiratory Capacity, Activity and Expression of OXPHOS Complexes that, Except for Complex V, Are Functionally Normal

**Figure 3:**
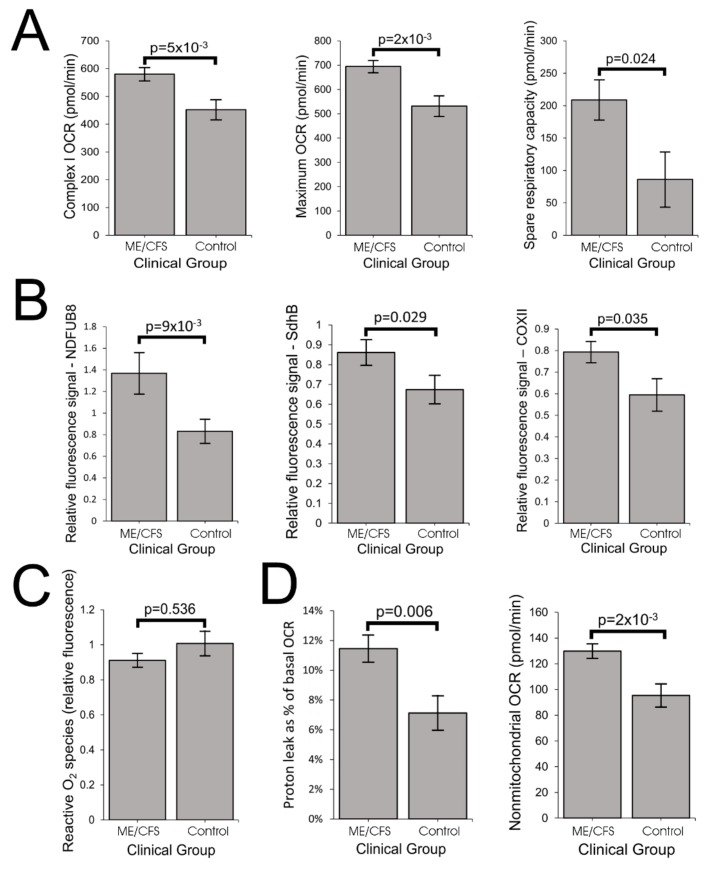
ME/CFS lymphoblasts exhibit elevated respiratory capacity and expression of oxidative phosphorylation (OXPHOS) complexes. Error bars are standard errors of the mean. (**A**) Complex I OCR, maximum OCR and spare respiratory capacity are elevated in ME/CFS lymphoblasts (independent *t*-test). The OCR was measured in lymphoblasts from ME/CFS and control individuals by the Seahorse XFe24 Extracellular Flux Analyzer. Each ME/CFS (*n* = 50) and control (*n* = 22) cell line was assayed over four replicates in each of at least three independent experiments. (**B**) Relative expression levels of Complex I subunit NDUFB8, Complex II subunit SdhB and Complex IV subunit COXII were elevated in semiquantitative Western blots (independent *t*-test). Each ME/CFS (*n* = 48) and control (*n* = 17) cell line was assayed in at least three independent experiments. (**C**) Intracellular ROS levels (relative background-subtracted Deep Red fluorescence) are unchanged in ME/CFS lymphoblasts (independent *t*-test). Each ME/CFS (*n* = 49) and control (*n* = 22) cell line was assayed in duplicate within each of at least three independent experiments. (**D**) Proton leak as % of basal OCR and the nonmitochondrial OCR are elevated in ME/CFS lymphoblasts (independent *t*-test). Each ME/CFS (*n* = 50) and control (*n* = 22) cell line was assayed over four replicates per experiment in at least three independent experiments.

To achieve normal ATP synthesis rates and steady state levels, ME/CFS lymphoblasts may compensate for the reduced efficiency of respiratory ATP synthesis by upregulating respiratory electron transport. This was demonstrated to be the case by the increased maximum OCR of the CCCP-uncoupled mitochondria and the main contributor to this, uncoupled O_2_ consumption by Complex I (rotenone-sensitive) as well as the spare respiratory capacity not utilized by basal respiration (Figure 3A, Figure S5 Panel 2A). This elevated respiratory capacity in ME/CFS mitochondria implies an increase in the expression, import or activity of the proteins in these complexes and in the supporting pathways. To determine if this was the case, we used semiquantitative Western blotting on crude lysates from ME/CFS and control lymphoblasts to assay the relative expression levels of indicative subunits of each of the five mitochondrial respiratory complexes. We found significant increases in the levels of Complex I, II, and IV subunits by semiquantitative Western blotting (Figure 3B, Figure S5 Panel 2B). Smaller increases in the levels of subunits in the other complexes (III, V) were not statistically significant (Figure S6).

**Figure 4:**
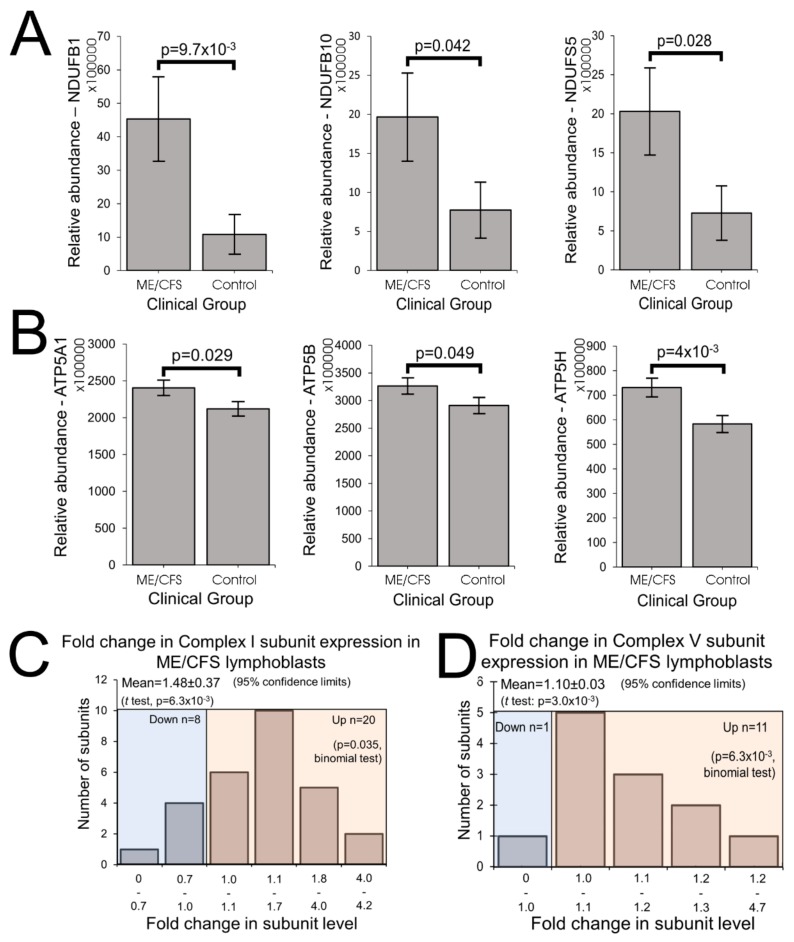
ME/CFS lymphoblasts exhibit elevated expression of Complexes I and V in their whole cell proteomes. (**A**) Complex I subunits NDUFB1, NDUFB10, and NDUFS5 were significantly upregulated (iBAQ) in whole cell proteomes (independent *t*-test). Each ME/CFS (*n* = 22) and control (*n* = 16) cell line was sampled once. (**B**) Complex V subunits ATP5A1, ATP5B, and ATP5H were significantly upregulated (iBAQ) in whole cell mass spectrometry proteomics experiments (independent *t*-test). Each ME/CFS (*n* = 22) and control (*n* = 16) cell line was sampled once. (**C**) Of the 44 known Complex I subunits, 31 were present amongst the 3700 proteins detected in the whole cell proteomes, 28 of these in both control and ME/CFS lymphoblast samples. Their relative abundances were compared and the majority were elevated in ME/CFS cells (fold change > 1) The background colors separate the two tested proportions in this data: blue corresponding to the samples with reduced abundance and pink for samples with elevated abundance. (**D**) 12 Complex V subunits were detected within the whole cell proteomes of ME/CFS and control lymphoblasts. Their relative abundances were compared to the controls and the majority were elevated in ME/CFS cells (fold change > 1). The background colors indicate the samples with reduced abundance (blue) and samples with elevated abundance (pink).

These results suggest that in ME/CFS cells the expression of the respiratory complexes is homeostatically increased to compensate for inefficient ATP synthesis. To test this hypothesis further, we conducted whole cell proteomics analysis of 16 control and 22 patient lymphoblast cell lines. For several of the individual subunits of both Complexes I and V, the measured increase in expression of that subunit alone exceeded the threshold for statistical significance (Complex I—Figure 4A, Figure S5 Panel 3A; Complex V—Figure 4B, Figure S5 Panel 3B). Thirty-one of the 44 Complex I subunits were detected, 26 of these in more than five samples and most exhibited increases in their levels in ME/CFS cells compared to the controls (Figure 4C, Table S1), significantly more than would be expected by chance. The average expression of the Complex I subunits in ME/CFS cells was significantly higher than in the controls (1.48 fold, *p* = 6.3 × 10^−3^). Eleven of the 12 Complex V subunits were upregulated, a significantly higher fraction than would be expected by chance (Figure 4D, Table S1). Average expression levels were also significantly elevated for the subunits of Complex V (1.1 fold, *p* = 3.0 × 10^−3^). Similar results for both complexes were found when the *t* test included only subunits detected in more than five samples (Table S1). Taking into account this 10% higher expression of Complex V subunits, the relative inefficiency of Complex V in the ME/CFS cells is even greater than measured directly by respirometry—almost 25% lower than the controls.

The expression of subunits of the other respiratory complexes was also higher in ME/CFS lymphoblasts in our proteomics analysis, but the increases did not reach statistically significant levels (Table S1). Although the small number of Complex II subunits did not provide sufficient statistical power to detect significant upregulation in this kind of test, the levels of each of the detected subunits appeared to be elevated, consistent with the significant increase detected by Western blotting. Together, these results show that the levels of the key mitochondrial OXPHOS proteins are elevated in ME/CFS cells, thereby explaining the increased maximum OCR in Seahorse respirometry assays.

Electron flow from Complex I through Complexes III and IV is the major contributor to respiratory electron transport, the contribution from Complex II in these cells being very small [21]. Having observed that Complex V was functioning inefficiently, contributing a smaller fraction to the basal respiration rate in ME/CFS lymphoblasts than in control cells, we determined whether Complexes I, III, and IV were functioning normally.

In contrast with the fractional contribution of Complex V to basal respiration, we found that the fractional O_2_ consumption by uncoupled electron flow from Complex I through Complexes III and IV to molecular oxygen was unchanged in the ME/CFS cells (Figure S7). Thus electron transport is functionally normally in ME/CFS lymphoblasts and the defect in oxidative phosphorylation is isolated to ATP synthesis by Complex V. This can be concluded since a defective Complex I, III, or IV would result in the electron flow through these complexes contributing proportionately less to total OCR, even if compensatory upregulation of expression were to bring respiration rates back to normal or even higher than normal absolute levels. We conclude that electron transport in the ME/CFS lymphoblasts is functionally normal, but elevated in capacity because of elevated levels of expression of the respiratory complex proteins involved.

Another indicator of abnormalities in electron transport is the level of reactive oxygen species (ROS). ROS are produced by electron “leakage” to molecular oxygen at the point where electrons are normally passed to Complex III from either Complex I or II. ROS production can be increased either by an increased flux of electrons through the electron transport chain or by a “downstream” blockage that diverts the electron flow. We therefore measured the levels of ROS in patient and control lymphoblasts and found no change in intracellular ROS levels in the ME/CFS cells when compared with controls (Figure 3C, Figure S5 Panel 2C). This is consistent with the insignificant changes in basal respiration rate and also suggests that the electron transport chain (ETC) is functionally normally.

The compensatory elevation of expression and uncoupled activity of mitochondrial respiratory complexes shown by our data suggests that ME/CFS mitochondria should exhibit increased capacity for proton motive force (PMF)-driven transport processes in the mitochondrial membrane that support and maintain mitochondrial biogenesis and function. For example, the levels of key proteins in the mitochondrial protein import complexes were elevated in the ME/CFS proteomes, as were the levels of multiple SLC25 transporters in the inner mitochondrial membrane (Figure S8). Such supporting transport pathways contribute to the so-called “proton leak”, which refers to depletion of the PMF by mitochondrial transport processes other than ATP synthesis by Complex V. As expected, the proton leak was significantly elevated as a proportion of the basal metabolic rate (Figure 3D, Figure S5 Panel 2D) in ME/CFS lymphoblasts when compared with controls. This result also confirms that Complex V in these ME/CFS cells is operating at a lower coupling efficiency, consuming relatively less of the mitochondrial respiratory O_2_ consumption while the proton leak consumes more.

The foregoing data shows that mitochondrial respiratory capacity in ME/CFS lymphoblasts is upregulated, perhaps in response to inefficient ATP synthesis by Complex V. This is coupled with increased depletion of energy by transport processes which we hypothesized would include those that provide oxidizable substrates to the mitochondria. We therefore expect the rates of nonmitochondrial catabolic processes that provide these substrates also to be increased. Our data supports such a possible shift in metabolism as the “nonmitochondrial” OCR, an indicator of catabolic rate, was significantly elevated in ME/CFS lymphoblasts (Figure 3D, Figure S5 Panel 2D).

### 2.4. Mitochondrial Abnormalities in ME/CFS Lymphoblasts Are Correlated with Disease Severity

**Figure 5:**
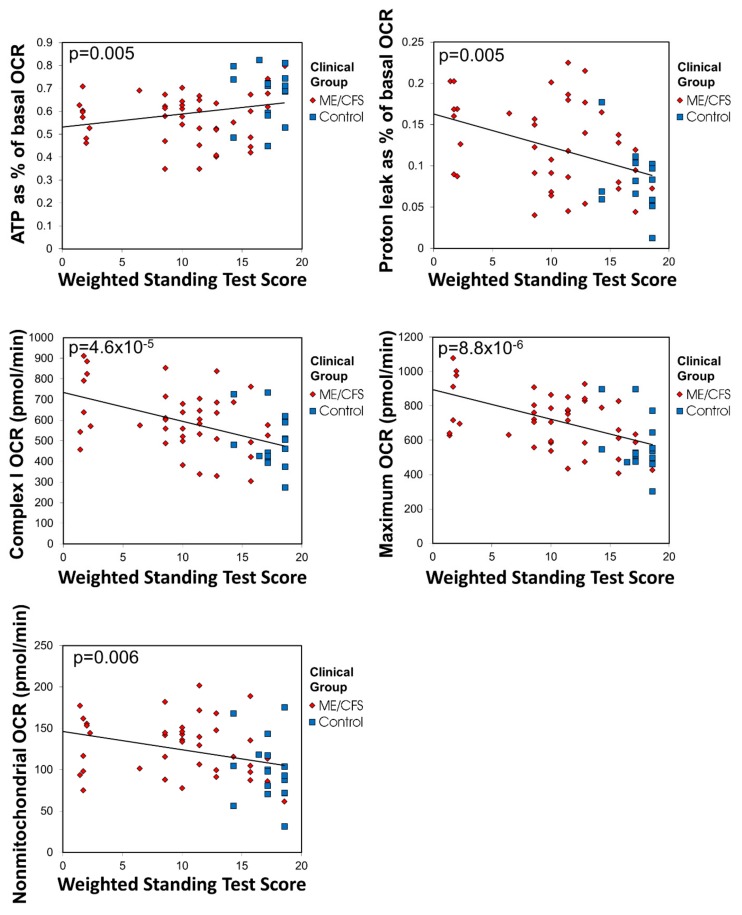
The respiratory shift in ME/CFS lymphoblasts is correlated with disease severity. ATP synthesis as a percentage of basal respiration and the elevated proton leak, maximum, nonmitochondrial and Complex I OCRs correlate with Weighted Standing Test score, a measure of disease severity in which lower values indicate more severe clinical presentation (Pearson correlation with indicated significance) (ME/CFS *n* = 45, control *n* = 17). Patients whose illness was so severe as to preclude them taking the test were not included. Lines fitted by linear least squares. Outliers falling outside 95% confidence limits were excluded.

In view of the foregoing functional abnormalities in ME/CFS mitochondria, we tested the key elevated parameters (Figure 5) for correlation with disease severity as assessed by the Richardson and Lidbury Weighted Standing Test [24]. We found that all were correlated with the clinical outcomes. This provides strong evidence for the idea that the mitochondrial abnormalities we found are clinically relevant.

### 2.5. Mitochondrial Genome Copy Number and Mass per Cell Are Unchanged in ME/CFS Lymphoblasts, but Mitochondrial Membrane Potential Is Lowered

**Figure 6:**
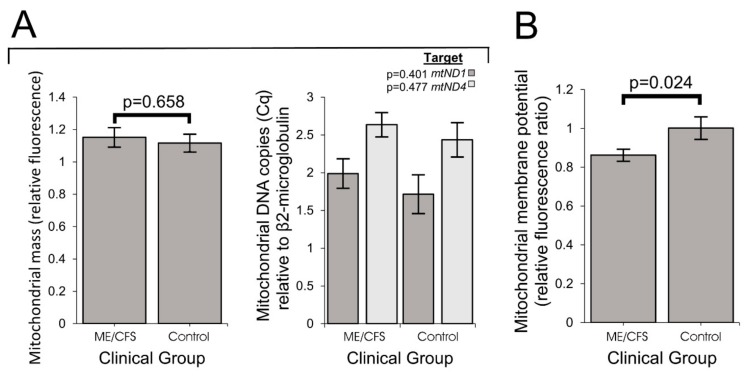
Mitochondrial genome copy number and mass per cell are unchanged in ME/CFS lymphoblasts, but mitochondrial membrane potential is lowered. Error bars represent standard errors of the mean. (**A**) Mitochondrial mass (background-subtracted Mitotracker Green fluorescence normalized to an internal control cell line) and genome copy number (qPCR of two mitochondrial genes, mtND1 and mtND4, relative to nuclear β2-microglobulin gene) are unchanged in ME/CFS lymphoblasts (independent *t*-test). Each ME/CFS (*n* = 50) and control cell line (*n* = 22) was assayed in duplicate within each of at least three independent experiments. (**B**) Mitochondrial membrane potential is significantly reduced in ME/CFS lymphoblasts (independent *t*-test). The relative mitochondrial membrane potential (Δψ_m_) was measured in lymphoblasts from ME/CFS and control individuals (ratio of MitoTracker^®^ Red CMXRos (Δψm-dependent) to MitoTracker^®^ Green (mitochondrial mass-dependent) fluorescence, normalized to an internal control cell line). Each ME/CFS (*n* = 50) and control (*n* = 22) cell line was assayed in duplicate within each of at least three independent experiments.

Mitochondrial mass as a measure refers to total inner mitochondrial membrane within the cell’s dynamic network of mitochondria [25], whilst membrane potential is the charge gradient across the inner mitochondrial membrane—which constitutes part of the total PMF (proton motive force), driving ATP synthesis by Complex V (ATP synthase). Since the expression of mitochondrial proteins is upregulated in ME/CFS lymphoblasts, it was possible that this would be reflected in an increase in the total cellular mitochondrial content. To assess this, we have assayed both the mitochondrial genome content relative to the nuclear genome, as well as the mitochondrial membrane “mass” per cell and observed no differences between ME/CFS and control lymphoblasts (Figure 6A, Figure S9 Panel 1A). This suggests that the ME/CFS mitochondria contain a higher concentration of mitochondrial respiratory proteins than the control cells.

The inefficiency of ATP synthesis by Complex V means that basal respiration rates by ME/CFS lymphoblast mitochondria would also be reduced, were it not for the compensatory upregulation of their respiratory complex levels. This allows them to maintain normal ATP synthesis rates and, as observed, is accompanied by increased respiratory capacity of the electron transport chain (mostly Complex I activity), supported by an increased use of the proton gradient to drive mitochondrial protein import and other mitochondrial membrane transport processes (the “proton leak”). These changes could result in a reduction of the steady state mitochondrial membrane potential, because of elevated “consumption” by the “proton leak”. To test the hypothesis that the Δψ_m_ was reduced in ME/CFS lymphoblasts, we measured Mitotracker Red fluorescence, whose binding to the mitochondrial membrane is Δψ_m_-dependent. This was normalized to the mitochondrial membrane “mass” (measured by MitoTracker^®^ Green fluorescence) and an internal control cell line to determine the relative Δψ_m_. We found that both Mitotracker Red fluorescence (*p* = 0.02) and the mitochondrial membrane potential (Δψ_m_) were reduced in ME/CFS lymphoblasts (Figure 6B, Figure S9 Panel 1B). Similar findings were recently reported for CD4^+^ and CD8^+^ T-cells [15].

### 2.6. Glycolytic Rates Are Unaffected in ME/CFS Lymphoblasts, but Levels of Enzymes Involved in Fatty Acid β-Oxidation and the TCA Cycle Are Elevated

**Figure 7:**
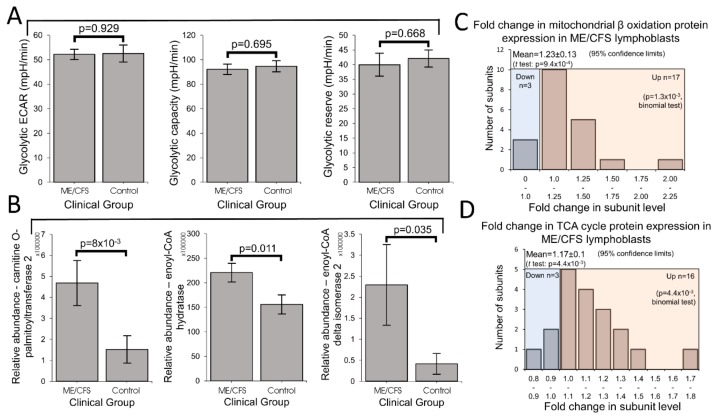
Glycolysis rates are unaffected in ME/CFS lymphoblasts, but levels of enzymes involved in fatty acid β-oxidation and the TCA cycle are elevated Error bars represent standard errors of the mean. (**A**) Glycolytic rate, capacity and reserve are unchanged in ME/CFS lymphoblasts (independent *t*-test). The ECAR was measured in lymphoblasts from ME/CFS and control individuals by the Seahorse XFe24 Extracellular Flux Analyzer. Each ME/CFS (*n* = 23) and control (*n* = 16) cell line was assayed over four replicates in at least three independent experiments. (**B**) Fatty acid carrier carnitine O-palmitoyltransferase 2 and β-oxidation enzyme enoyl-CoA delta isomerase 2 were significantly upregulated (iBAQ) in whole cell mass spectrometry proteomics experiments (independent *t*-test). Each ME/CFS (*n* = 22) and control (*n* = 16) cell line was sampled once. (**C**) 20 proteins involved in mitochondrial β-oxidation were detected within the whole cell proteomes of ME/CFS and control lymphoblasts. Their relative abundances were compared to the controls and the majority were elevated in ME/CFS cells (fold change > 1). The background colors separate the two tested proportions in this data: blue corresponding to the samples with reduced abundance and pink for samples with elevated abundance. (**D**) 19 proteins involved in the TCA cycle were detected within the whole cell proteomes of ME/CFS and control lymphoblasts. Their relative abundances were compared to the controls and the majority were elevated in ME/CFS cells (fold change > 1). The background colors indicate the samples with reduced abundance (blue) and samples with elevated abundance (pink).

Published metabolomics work suggests that ME/CFS metabolism is dysregulated such that the provision of acetyl CoA to the TCA cycle by glycolytic pyruvate is reduced and there is instead a shift away from carbohydrate metabolism towards alternative oxidizable substrates [16]. Other work suggests that glycolysis itself is unimpaired and that reduced conversion of pyruvate to acetyl CoA by PDH is responsible for a similar downstream consequence [17]. However, Tomas et al. (2017) found no difference in glycolysis between lymphocytes from ME/CFS and control individuals [8], while Nguyen et al. (2018) reported a reduction in glycolytic capacity in a small sample of NK cells from ME/CFS patients [26]. Most recently, glycolytic rates were reported to be reduced in ME/CFS CD4^+^ and CD8^+^ T-cells [15]. To investigate glycolysis in intact ME/CFS lymphoblasts, we modified and implemented an optimized Seahorse assay to assess real-time glycolytic production of lactate in live cells by measuring the extracellular acidification rate (ECAR) of the medium. We found no difference between ME/CFS and control cells in glycolytic rate, reserve or capacity (Figure 7A, Figure S9 Panel 2A).

Previous metabolomic studies have suggested that lipid metabolism is dysregulated in ME/CFS [16,27,28,29]. Since the elevated “proton leak” and “nonmitochondrial” OCR were consistent with an increase in mitochondrial uptake and catabolism of alternative oxidizable substrates, we investigated the expression of mitochondrial fatty acid transporters enzymes responsible for fatty acid β-oxidation in whole cell proteomes of ME/CFS and control lymphoblasts. We found proteins in both groups to be significantly elevated in the ME/CFS cells (Figure 7B,C; Figure S9 Panel 2B). The levels of all three fatty acid transport proteins, carnitine acyltransferase I and II as well as the acyl carnitine carrier protein, were elevated, although this reached statistical significance only in the case of carnitine acyl transferase II. Furthermore, 17 of 20 proteins involved in mitochondrial β oxidation were upregulated (binomial test, *p* = 2.6 × 10^−3^), with average levels 23 ± 13% higher than controls (*t* test, *p* = 9.4 × 10^−4^). All six of the central enzymes in the mitochondrial β oxidation cycle were upregulated (binomial test, *p* = 0.016) and the level of one of these (enoyl-CoA hydratase) was significantly elevated on its own. If the rates of mitochondrial β oxidation were elevated in ME/CFS cells in accordance with the expression of the proteins involved, these pathways would provide acetyl CoA to the TCA cycle at faster rate. In keeping with this, we found that the expression of TCA cycle proteins was also elevated in ME/CFS lymphoblasts (Figure 7D). Thus, 16 of 19 detected proteins involved in the TCA cycle were more abundant in the ME/CFS proteome (binomial test, *p* = 2.2 × 10^−3^). The mean expression level of TCA cycle proteins in ME/CFS lymphoblasts was 17 ± 10% higher than in the control cells (*t* test, *p* = 1.0 × 10^−3^). Taken together, these results show that ME/CFS lymphoblasts have an increased capacity for using mitochondrial β oxidation to drive oxidative phosphorylation at rates faster than would otherwise have been the case.

### 2.7. Stress-Sensing Pathways in ME/CFS Are Perturbed—TORC1 Is Chronically Hyperactivated

**Figure 8:**
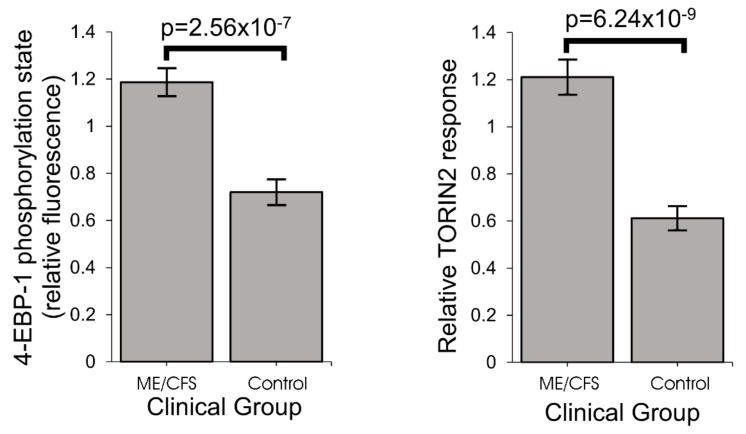
Stress sensing pathways in ME/CFS are perturbed—TORC1 (Target of Rapamycin Complex I) is chronically hyperactivated. Error bars represent standard errors of the mean. TORC1 activity and response to TORIN2. inhibition is elevated in ME/CFS lymphoblasts (independent *t*-test). Each ME/CFS (*n* = 45) and control (*n* = 22) cell line was assayed over at least three independent experiments. Data is expressed in relative terms as each experiment is normalized to an internal control cell line.

The compensatory action to bring ATP levels back to normal in ME/CFS lymphoblasts despite Complex V’s inefficiency is likely to be driven by the signaling networks that sense and homeostatically respond to diverse cellular stresses [30,31,32,33,34,35]. A central element in these pathways, interconnected with the others, is the protein kinase, TORC1 (Target of Rapamycin Complex I), which coordinates the translational upregulation of major functional groups of proteins, including nuclear-encoded mitochondrial proteins. We measured its activity in ME/CFS and control lymphoblasts by assaying the phosphorylation state of one of its key substrates, 4E-BP1 (eukaryotic translation initiation factor 4E-Binding Protein 1) [36]. 4E-BPs are phosphorylated by TORC1, whose catalytic subunit is mTOR (mechanistic Target Of Rapamycin). Together with S6 kinase (S6K), 4E-BP1 mediates the roles of TORC1 in regulating translation in the cytosol of mRNAs encoding major functional groups of proteins [37,38,39]. As one of the key substrates of TORC1 involved in regulating protein synthesis, 4E-BP1 is often used as a marker of TORC1 activity [40]. We found that 4E-BP1 phosphorylation levels were significantly elevated in ME/CFS lymphoblasts and were accompanied by a correspondingly increased response to the mTOR inhibitor, Torin2 (Figure 8, Figure S10). This chronic elevation of TORC1 activity could explain the increased expression of mitochondrial proteins and respiratory capacity that we found in ME/CFS lymphoblasts [36,40].

## 3. Discussion

Previous steady state measurements and metabolic flux measurements of mitochondrial respiratory function in ME/CFS lymphocytes have suggested that in ME/CFS cells there is either a generalized reduction [6,7,8] or little change [9,15] in mitochondrial activity and respiratory capacity. However, functionally normal OXPHOS Complex I to IV activity has also been reported in ME/CFS lymphocytes [11,41], while the expression of mitochondrial proteins is upregulated in patient saliva, platelets and lymphocytes [12,13,23]. Elevated nonmitochondrial ATP production has also been reported in ME/CFS lymphocytes [11].

In this work we have resolved these inconsistencies by revisiting the issue of mitochondrial function and capacity in immortalized lymphocytes (lymphoblastoid cell lines or lymphoblasts). Although ex vivo lymphocyte populations are well suited for rapid characterization of relatively stable molecular features, such as the patterns of cell surface antigens they express, they may be less well suited to studying rapidly labile processes such as metabolism, mitochondrial function or intracellular signaling activities. As we showed here, unactivated ex vivo lymphocytes are metabolically quiescent and dying. A difference between patient and control groups in the depth of this quiescence and/or the extent of cell death may thus explain the previously reported reduction in mitochondrial activities in ME/CFS lymphocytes. One recent study examined mitochondrial respiratory function in resting and stimulated CD4^+^ and CD8^+^ T-cells from ME/CFS subjects and controls [15]. The only significant differences found were small reductions in the ME/CFS CD8^+^ lymphocytes in the proton leak in resting and ATP synthesis in stimulated cells. Surprisingly however, the overnight stimulation (with anti-CD3/anti-CD28 beads plus IL2) made almost no difference to mitochondrial respiratory function in these cells. This suggests that under the conditions used, the mitochondria in these cells were still quiescent after the overnight activation stimulus.

We found that not only were PBMCs quiescent, but the fraction of dead cells after 1–3 days incubation in culture medium was dramatically greater for ME/CFS lymphocytes than for control lymphocytes. It is likely that in some previous studies, the ME/CFS lymphocytes assayed for mitochondrial activity included a higher proportion of dead cells than did the controls. Tomas et al. (2017) found no significant difference in the viability of fresh and frozen PBMCs and no difference between ME/CFS and control samples [8], a result that accords with our regression analysis of the death rates of PBMCs in culture (Figure 1). However, these authors followed the common procedure in their Seahorse assays of incubating the cells overnight in situ in the assay plates before the respirometry measurements were done. Although they did not detect significant differences between frozen and fresh PBMCs in the Trypan Blue staining *before* assay, they did observe consistently significant reductions in respiration of frozen compared to fresh cells, again *after* overnight incubation in assay medium. Our results suggest that by this time there may have been a significant fraction of dead cells both in their control and ME/CFS samples and that this fraction may have been higher in the ME/CFS samples. The greater mortality rates for ME/CFS lymphocytes are not surprising given that pharmacological inhibition of mitochondrial respiration, including Complex V impairment, has long been known to result in apoptotic cell death in ex vivo lymphoid cells [42].

By contrast, the lymphoblastoid cell lines (LCLs or lymphoblasts) used in this work are metabolically active lymphoid cells that may better represent activated lymphocytes, which drive inflammation in vivo [43]. The use of cultured lymphoblasts in this way as models of not only metabolically active lymphoid but other cell types, both in health and disease, has been reviewed in detail previously [44,45]. Because the viral genome exists in multiple copies as a circular episome in the latent state in infected cells, it does not disrupt the genome, but affects gene expression patterns in favor of proliferation, as does B cell activation by other means. Lymphoblasts have thus been used both in genetic and genomic studies, including the well-known 1000 Genomes Project [46]. They have also been used, as here, in functional studies of diverse, complex diseases, including autism, schizophrenia, Alzheimer’s, and Parkinson’s disease [21,45]. Whereas PBMCs, used ex vivo in some other ME/CFS studies, are a complex mixture of cell types, EBV-mediated immortalization and culture selects B cells from this population, because the EBV receptors are expressed in B cells. It has been reported previously that the cell type composition of PBMCs from ME/CFS patients is different from healthy controls [47] and such differences could also contribute to observed functional differences in the mixed populations in ex vivo PBMCs. We chose not to clone our cell lines, but as a result of the selective nature of EBV infection, they can be expected to be more homogeneous in cell type than the PBMCs from which they were selected.

As EBV-infected B cells begin to proliferate, around 250 genes become hypomethylated [48]. Many of these genes already exhibit low levels of methylation and high levels of expression in resting B cells, while many others exhibit hypomethylation and overexpression during B cell proliferation. They fall into 6 major Gene Ontology (GO) categories—immune response, homophilic cell adhesion, humoral immune response, B cell receptor signaling pathway, inflammatory response and chemotaxis genes. In the whole cell proteomes of lymphoblasts from both healthy and ME/CFS individuals, we observed expression of many B-cell specific proteins whose expression was not significantly different in ME/CFS and control cell lines (data not shown). The overall pattern of expression is consistent with lymphoblasts having had their normal B cell transcription and proliferation program activated by the virus [48]. Importantly, this phenotype is stable through up to 180 cell doublings, unlike other in vitro methods of B cell activation (such as antigen stimulation) which induce similar changes in gene expression that increase and decline over only a few days and culminate in cell death.

Like primary cell cultures of other cell types such as myoblasts (which die after a few doublings) or fibroblasts (which progressively enter senescence between *ca.* 30–60 doublings), lymphoblasts eventually die off after *ca.* 160–180 doublings [45]. Thereafter, the original lymphoblast population may be replaced by cells bearing mutational changes in the genome that support ongoing proliferation. At this point the cells may no longer be representative of activated forms of the B lymphocytes from which they were derived. In the work reported here, none of the lymphoblast cultures were allowed to proceed through more than a handful of cell doublings before storage or use in experiments.

During their more limited life spans, primary cell lines like myoblasts and fibroblasts undergo a progressive process called replicative senescence as part of which their relevant phenotypic and molecular features may change [49]. Fibroblast and myoblast gene expression programs are also tightly regulated by contact inhibition so that their phenotypes can be dramatically affected by their density in culture [50]. Furthermore, their life span and phenotypes may be affected by the age of the donor from whose tissues they were derived. These various sources of phenotypic differences can be disentangled but this requires care, appropriate controls and may need larger samples or more experiments to account for the additional variables. Provided long periods of culture approaching their replicative life span limits are avoided, lymphoblasts do not present these problems. They are not contact-inhibited and their metabolic phenotypes are stable in culture and storage, depending only, as we have shown here for ME/CFS and previously for Parkinson’s disease [21], on the disease state, not the gender or age of the person from whom they were isolated. Despite these advantages, it will be important in future work to determine whether the mitochondrial and cell signaling anomalies in ME/CFS lymphoblasts are also observed in other cell types.

Our results showed that ME/CFS lymphoblasts exhibit an isolated Complex V inefficiency that is accompanied by upregulation of mitochondrial protein expression, including mitochondrial respiratory complexes and enzymes involved in the TCA cycle, fatty acid uptake and β-oxidation. These findings confirm that these ME/CFS cells do indeed exhibit a mitochondrial deficiency in ATP generation, but reveal that, in lymphoblasts at least, this specifically involves Complex V rather than a generalized reduction in all mitochondrial functions. This profile of mitochondrial dysfunction in intact ME/CFS lymphoblasts is distinct from the mitochondrial hyperactivity we previously found in Parkinson’s disease lymphoblasts [21], so cannot be a simple reflection of neuroinflammatory processes believed to occur in both diseases.

What might cause such a mitochondrial Complex V inefficiency? Three possibilities are a mutation affecting one of the Complex V subunits or assembly proteins, an elevation of the relative use of the proton motive force for other purposes (“proton leak”) making less available for ATP synthesis or a dysregulation of Complex V itself. A mutational defect in Complex V seems unlikely in view of the failure of previous investigations to uncover any single nucleotide polymorphisms in Complex V genes that associate with the disease state [51,52]. The second possibility, that Complex V is inefficient in these ME/CFS cells because of the elevated use of the proton motive force by other processes, is suggested by the elevated proton leak we measured in ME/CFS lymphoblasts. However, the ME/CFS mitochondria have excess unused respiratory capacity and respiratory complex levels. These would indicate that the electron transport capacity in ME /CFS lymphoblasts is more than sufficient to allow Complex V to operate at normal efficiency. Dysregulatory inhibition of Complex V is a third possibility. It is known that mitochondrial ATP synthase activity can be regulated by a variety of proteins, small molecules and signaling pathways, some of them by acting through Complex V’s own inhibitory subunit AIF1 [53,54]. These possible causes for Complex V inefficiency in ME/CFS lymphoblast mitochondria should be investigated in future work.

It is possible that the dysregulation of Complex V and mitochondrial function in ME/CFS lymphoblasts arises because they respond differently to EBV infection than do control lymphoblasts. We showed that the EBV genome copy number did not differ between the participant groups and, in any case, had no effect on any of our assays. Nevertheless, it is possible that in ME/CFS lymphoblasts, EBV reactivates more readily to enter the lytic cycle and this in turn affects mitochondrial function. This is an attractive idea that, if true, would raise the possibility that EBV may also reactivate more readily in infected ME/CFS B-cells in vivo. Despite the appeal of this hypothesis, the profile of mitochondrial changes we observed is not consistent with the reported effects of EBV on mitochondria in the lytic cycle. These include *decreases* in ROS production, mitochondrial membrane potential, expression of mitochondrial proteins, and mitochondrial biogenesis [55,56]. Although we did observe decreased mitochondrial membrane potential in ME/CFS lymphoblasts, we found no change in ROS levels, no change in mitochondrial mass or genome copy number and an elevation not a reduction in expression of mitochondrial proteins. We are unaware of any evidence that EBV causes an isolated inefficiency in Complex V coupled with an elevated proton leak and an increase in maximum OCR and Complex I activity. Instead of EBV reactivation causing mitochondrial dysfunction in ME/CFS lymphoblasts, it is also possible that the reverse occurs—namely that mitochondrial dysfunction causes the virus to be more readily reactivated. This possibility is suggested by the fact that EBV is reactivated by cellular stress and could potentially contribute to the post-exertional malaise that characterizes ME/CFS. The interactions between EBV and mitochondrial function in B-cells and lymphoblasts from ME/CFS patients are clearly worth pursuing in future work.

The elevated maximum respiratory capacity, mitochondrial Complex I activity, and proton leak we found in ME/CFS lymphoblasts are consistent with the higher mitochondrial protein expression we observed in these cells, as assayed using both semiquantitative Western blots and whole cell proteomics. Consistent with this, it was reported previously that the expression of genes encoding mitochondrial proteins is upregulated in ME/CFS saliva, lymphocytes, and platelets [12,13,23], while increased translocase activity into the mitochondrial matrix has also been reported [7]. Together our results suggest a model in which the Complex V defect is a proximal activator of compensatory upregulation of expression of mitochondrial proteins.

The increased expression of diverse mitochondrial proteins in ME/CFS observed by us and others [11,12,13] suggests the possibility that mitochondrial biogenesis more broadly is activated in these cells. However, we found that the mitochondrial membrane “mass” per cell (Mitotracker Green fluorescence) and the copy number of the mitochondrial genome relative to the nuclear genome were unchanged in the ME/CFS lymphoblasts. Accordingly, the mitochondria in these cells appear to have higher concentrations of mitochondrial respiratory proteins and catabolic enzymes.

One of the key upstream regulators of mitochondrial protein expression is TOR Complex I (TORC1 whose catalytic subunit is mTOR, the mechanistic Target Of Rapamycin). We found that TORC1 activity is elevated in ME/CFS lymphoblasts. The expression of mitochondrial enzymes involved in electron transport is known to be upregulated by TORC1 via selective activation of translation via inhibitory phosphorylation of the TORC1 target 4E-BP1 [20]. In addition to its actions on the translation of nuclear-encoded mitochondrial proteins, TORC1 upregulates the expression of transcription factors PGC-1α (transcriptionally via Yin Yang 1) and TFAM (translationally), which respectively induce the transcription of nuclear and mitochondrial genes encoding mitochondrial proteins [19]. Most notable amongst the mitochondrial proteins whose translation is upregulated by TORC1 are nuclear-encoded subunits of Complexes I and V [20], the two respiratory complexes whose expression we found to be the most evidently elevated in the whole cell proteomes of ME/CFS lymphoblasts.

Mitochondrial enzymes involved in the β oxidation of fatty acids are amongst the many proteins whose expression is upregulated by PGC1α and thus indirectly by TORC1. Fatty acid β oxidation provides acetyl CoA to the TCA cycle, as does glycolysis, but it yields more ATP per oxidized carbon than does glycolysis. This also makes thermodynamic sense, given the more highly reduced state of the carbons in fatty acid chains compared to those in sugar molecules. ME/CFS cells could shift their metabolism in favor of fatty acid β oxidation because of a deficiency in glycolysis [15,16] or pyruvate dehydrogenase which partially oxidizes pyruvate and supplies acetyl CoA to the TCA cycle [17]. It has also been suggested that in ME/CFS cells metabolism shifts away from glycolysis in favor of the pentose phosphate pathway which can oxidize sugars in the cytosol to generate reducing power that can be transported into the mitochondria to drive electron transport [28]. Our results support the existence of a metabolic shift, but we found no deficiency in glycolysis rates, glycolytic reserve or glycolytic capacity. Although there have been reports of reduced rates of glycolysis in ME/CFS lymphocytes [26], this contrasts with reports by others [8]. Instead of impaired glycolytic capacity driving the shift in metabolism, our results suggest that the change may be driven by elevated usage of alternatives such as the β oxidation of fatty acids. Fatty acid utilization rates were previously reported to be unchanged in permeabilized ME/CFS lymphocytes [41]. However, the permeabilization process (with loss of cytoplasmic cellular context, e.g., metabolites) or the metabolic quiescence and greater death rates of ME/CFS lymphocytes may have obscured the result in this previous study. It would be valuable in future experiments to measure fatty acid utilization rates in ME/CFS and control lymphoblasts.

TORC1 is not the only cellular stress sensing protein that regulates expression of proteins involved in cellular metabolism and mitochondrial function. It acts in concert with AMPK as part of a complex stress-sensing network [31,35]. AMPK is activated by a variety of cellular stressors including ATP insufficiency, elevated cytosolic Ca^2+^ concentrations and oxidative stress [57,58]. As the primary ATP sensor [59], AMPK is implicated in mitochondrial disease [60], and activates a variety of catabolic pathways that provide alternative oxidizable substrates to the mitochondria—including amino acids or fatty acids [61]. In ME/CFS lymphoblasts, with their chronically inefficient ATP synthesis, AMPK is expected to be chronically activated and participate in the compensatory upregulation of mitochondrial respiratory capacity that we observe. However, despite lower steady state ATP levels, the AMPK activation state was not significantly different between cultured muscle cells from CFS patients (Fukuda criteria) and healthy controls [62]. Future work should therefore test the hypothesis that AMPK is chronically activated in ME/CFS lymphoblasts and other cell types.

## 4. Materials and Methods

### 4.1. Participant Cohort

All participants were of European descent and belonged to two groups: ME/CFS patients (*n* = 51, 86% female, median age 50, age range 26–70) or healthy controls (*n* = 22, 68% female, median age 41, age range 21–58) without any family history of ME/CFS or similar myalgias, nor cohabiting with ME/CFS patients. There was no significant difference between the patient and control groups in either the gender proportions (Fisher’s exact test, *p* = 0.21) or age distribution (Fisher’s exact test, participants grouped by ages in 5 year increments, *p* = 0.19). Neither gender (ANOVA) nor age (multiple regression, Figure S2) had an effect on any of the parameters of mitochondrial function and TORC1 signaling (*p* > 0.05).

Lymphoblastoid cell lines (lymphoblasts) were isolated from all participants. Multiple assays were conducted on both lymphoblasts and the lymphocytes from which they were derived (peripheral blood mononuclear cells, PBMCs). However, because of limited PBMC supplies, subsets of the participant cohort were used for the tests using lymphocytes:

PBMC respirometry assays. The sample selection was determined by the availability of sufficient PBMCs for the experiments. This subcohort included 14 ME/CFS patients (71% female, median age 59, age range 38–71) and nine healthy controls (67% female, median age 41, age range 21–52). The gender proportions were not different (Fisher’s exact test *p* = 1.0), but the age distributions were (four control but no ME/CFS individuals under 30; seven ME/CFS individuals over 60; Fisher’s exact test *p* = 0.013 using 15-year bins). Neither age (multiple regression) nor gender (ANOVA) had any influence on the lymphocyte O_2_ consumption rates in either patients or controls (*p* > 0.05).

Lymphocyte cell death assays used samples from 35 ME/CFS individuals (89% female, median age 52, age range 26–71) and 14 control individuals (71% female, median age 42, age range 21–58). Neither the gender proportions (Fisher’s exact test *p* = 0.15) nor the age distributions were significantly different (Fisher’s exact test *p* = 0.13 using 15-year bins). Neither age (multiple regression) nor gender (ANOVA) had any influence on the lymphocyte death rate in either patients or controls (*p* > 0.05).

We used a randomly selected subset of lymphoblastoid cell lines to determine if the copy numbers of the EBV genome were different in patient and control groups and if they had any effect on the mitochondrial and cell signaling abnormalities we observed. This subset included 13 MECFS patients (85% female, median age 43, age range 26–62) and 15 controls (53% female, median age 41, age range 21–58). Neither the difference in gender proportions (Fisher’s exact test *p* = 0.11) nor the distribution of ages (Fisher’s exact test, *p* = 0.8 using 15 year bins) was statistically significant. Neither age (multiple regression) nor gender (ANOVA) had any influence on the EBV genome copy number in either patients or controls (*p* > 0.05).

A subset of the lymphoblast cell lines was also used for the proteomics analysis. It included 22 ME/CFS patients (90% female, median age 56, age range 26–71) and 16 controls (56% female, median age 39, age range 21–58). The difference between the patient and control groups in gender proportions (Fisher’s exact test *p* = 0.02) and age distribution (Fisher’s exact test, *p* = 0.03 using 10 year bins) was statistically significant. However, there was no significant effect of either age (multiple regression) or gender (ANOVA) on expression of the analyzed mitochondrial proteins in either patients or controls (*p* > 0.05).

Participants were assessed and samples collected at CFS Discovery Clinic, Melbourne, Australia, who have a long running specialization and interest in ME/CFS. Patients were diagnosed using the Canadian Consensus Criteria [63], assessed for postorthostatic tachycardia syndrome comorbidity, and asked to complete the Depression, Anxiety, and Stress Scale questionnaire and the Epworth Sleepiness Scale questionnaire. ME/CFS specific severity assessments were also conducted using Richardson and Lidbury’s Weighted Standing Time [24]. 15 mL of blood was taken per participant in heparin-treated vacutainer tubes (BD). Subjects with other known reasons for fatigue were excluded.

The project was approved by the Australian National University Human Research Ethics Committee (Reference 2015/193) and accepted as an externally approved project by the La Trobe University Human Ethics Committee (26 February 2016).

### 4.2. PBMC Isolation from Blood Sample and Immortalization

PBMCs were isolated from blood, stored and immortalized to form lymphoblast cultures as previously described [21]. Briefly, lymphocytes were isolated by Ficoll-Paque density centrifugation and counted. Next, 5 × 10^6^ cells were harvested for immortalization and were resuspended in 5 mL RPMI 1640 without L-glutamine (Life Technologies) supplemented with 1× Glutamax (Life Technologies), 10% FBS and 1% Penicillin/Streptomycin. Excess lymphocytes were separated into aliquots of 5 × 10^6^ cells, harvested and resuspended in 250 μL of Recovery™ Cell Culture Freezing Medium (Life Technologies) and stored at −80 °C.

For immortalization, 1 mL culture supernatant from B95.8 cells expressing Epstein–Barr virus (EBV) was added, and 150 µL of the mix was seeded per well in a 96-well U-bottom plate, then incubated for one hour within a humidified 5% CO_2_ incubator at 37 °C. A final concentration of 500 ng/mL Cyclosporin A (Sigma) was then added to each well. Cultures were fed weekly by replacing half of the medium with the same formulation, without disturbing the cells. This process was repeated over a period of approximately three weeks until the cells were confluent and growing rapidly, after which the lymphoblast cultures were processed as described in the following section.

### 4.3. Lymphoblast Cultures

Confluent lymphoblasts were transferred to T25 flasks in growth medium (Minimum Essential Medium α (Life Technologies), supplemented with 10% FBS and 1% Penicillin/Streptomycin) where they were cultured within a humidified 5% CO_2_ incubator at 37 °C. Cells were seeded at concentrations of no less than 2 × 10^5^ cells/mL, fed at intervals not exceeding three days by replacing 1/3 of medium with new medium, or split in a 1:3 ratio of cell culture to fresh medium as required. For storage, flasks containing confluent cultures were transferred to tubes, harvested by centrifugation, resuspended by 250 µL aliquots in Recovery™ Cell Culture Freezing Medium and stored at −80 °C. Dozens of lymphoblast aliquots per individual were stored to allow for ongoing access to cultures with low passage numbers for future use. Cells were removed from storage by thawing in a 37 °C water bath, harvested by centrifugation, resuspended in growth medium, and transferred to a fresh T25 flask.

Prior to commencing experiments, lymphoblast lines were cultured over as short a time and as few passages (2–5) as possible. For a set of triplicate, independent experiments, harvest, assay, and conduct of experiments occurred over approximately one week. As previously, two immortalized lymphoblast cell lines created from healthy donor blood were utilized as internal controls to normalize for variation between experiments where appropriate [19].

### 4.4. Viable Cell Counts

Lymphoblast or lymphocyte (PBMC) viable counts for all applications were determined by staining with Trypan Blue (Thermo-Fisher Scientific) prior to hemocytometer cell counting. Trypan Blue-stained cells were counted as dead and unstained, intact cells as viable. For the unimmortalized lymphocyte viability measurements over time, frozen aliquots were thawed in a 37 °C water bath, pelleted at 1000× *g* for 2 min and resuspended in 1 mL RPMI 1640 without L-glutamine supplemented with 1X Glutamax, 10% FBS and 1% Penicillin/Streptomycin. The cells were then washed at 1000× *g* for 2 min and resuspended in fresh medium of the same formulation. They were then seeded in 96-well U-bottom plate at a density of 1 × 10^6^ cells/mL, and kept in a humidified 5% CO_2_ incubator at 37 °C over the course of the experiment.

### 4.5. Mitochondrial Mass and Mitochondrial Membrane Potential (MMP)

Mitochondrial mass and MMP were assayed with modifications as previously using the mitochondrial dyes MitoTracker^®^ Green FM (Life Technologies) and MitoTracker^®^ Red CMXRos (Life Technologies) [21,64]. Both dyes bind specifically to mitochondrial membranes, MitoTracker^®^ Red binding being membrane potential (Δψ_m_)-dependent, while MitoTracker^®^ Green binding is not. Mitotracker Green fluorescence thus measures mitochondrial membrane “mass” and Mitotracker Red provides a measurement of Δψm when normalized to the Mitotracker Green signal.

Cells (7 × 10^5^) were harvested at 500× *g* for 5 min, and 1 × 10^5^ cells were plated per well into six wells of a 96 well black, clear flat bottom plate (Corning). The plate was incubated for 1 h at 37 °C with 5% CO_2_. To duplicate wells for each dye treatment, MitoTracker^®^ Green and Red were added to final concentrations of 200 nM, PBS added to background wells and Hoechst 33342 Nuclear Stain (Enzo Life Sciences) was included in every well at a final dilution of 1/2000, for normalizing under each treatment condition (excitation 355 nm, emission 455 nm). The plate was then incubated for 1 h at 37 °C and 5% CO_2_, the supernatant removed via aspiration and replaced with PBS. Fluorescences were read using the BMG Labtech Clariostar microplate reader. Relative mitochondrial mass was determined by background-subtracted MitoTracker^®^ Green FM fluorescence at excitation 470 nm and emission 515 nm, normalized to the background subtracted signal from the same number of cells of the internal control cell line. The MMP was determined from the background-subtracted MitoTracker^®^ Red CMXRos fluorescence (excitation 570 nm, emission 620 nm) divided by the background-subtracted fluorescence of MitoTracker^®^ Green FM.

### 4.6. Mitochondrial Stress Test (Seahorse Respirometry)

Oxygen consumption rates (OCR) of 8 × 10^5^ viable PBMCs or lymphoblasts per well were measured using the Seahorse XFe24 Extracellular Flux Analyzer with Seahorse XF24 FluxPaks (Agilent Technologies, Chicopee, Canada). Immortalized lymphoblasts were cultured in 3 mL growth medium per well in 6-well Costar plates prior to Seahorse experiments while PBMCs were recovered from storage and inoculated immediately. Seahorse assays were carried out as previously described in detail [21] and illustrated in Figure S3. Oxygen consumption rates (OCR in pmol/min) were measured (basal OCR) prior to and after successive injection of 1 µM oligomycin (ATP synthase inhibitor), 1 µM CCCP (carbonyl cyanide m-chlorophenyl hydrazone, an uncoupling protonophore), 1 µM rotenone (Complex I inhibitor) and 5 µM antimycin A (Complex III inhibitor). From the resulting data we determined the OCR associated with respiratory ATP synthesis (oligomycin-sensitive), the maximum OCR in CCCP-uncoupled mitochondria and the rotenone-sensitive OCR attributable to uncoupled Complex I activity, the antimycin-sensitive Complex II/III activity, the OCR by mitochondrial functions (e.g., protein import) other than ATP synthesis that are Δψm-driven (so-called ‘proton leak’), non-respiratory oxygen consumption (e.g., by cellular and mitochondrial oxygenases and oxidases), and the respiratory ‘spare-capacity’ (excess capacity of the respiratory electron transport chain that is not being used in basal respiration).

### 4.7. Glycolytic Stress Test

The extracellular acidification rate (ECAR) of live, intact lymphoblasts was measured using a modified glycolytic stress test in the Seahorse XFe24 Extracellular Flux Analyzer with Seahorse XF24 FluxPaks (Agilent Technologies, Chicopee, Canada). Immortalized lymphoblasts were cultured in growth medium in 6-well plates prior to Seahorse experiments.

In order to measure the ECAR of cells using this method, they must be firmly adhered to and evenly spread across the bottom of the assay plate wells. To achieve this, the Cell Culture Microplate was prepared as previously described with a Matrigel coating in the bottom of each well [21]. The plate was then left to dry at room temperature under laminar flow, and 8 × 10^5^ cells/well were later plated out in XF base medium (Agilent Technologies, Chicopee, Canada) containing 200 mM L-glutamine and 5 mM HEPES, as recommended by the manufacturer for glycolytic assays.

The sensor cartridge apparatus was rehydrated one day in advance by adding 1 mL XF Calibrant to each well and incubating at 37 °C until needed. The injection ports of the sensor cartridge apparatus were loaded with the following drugs, in chronological order of four injections to give the indicated final concentrations in the wells: Glucose-10 mM, Oligomycin-2 µM, Rotenone-1 µM and Antimycin A-5 µM (combined injection), 2-Deoxyglucose-50 mM. The treatment with the rotenone/antimycin combination allowed assessment of the impact of electron transport on ECAR by respiratory acidification coupled to passage of some glycolytic pyruvate through the TCA cycle to supply respiration.

Before and after each successive drug addition, the ECAR was measured over three time points, consisting of a 3 min mix, 2 min wait, and 3 min measurement time. These measurements were subsequently analyzed to determine the magnitudes of various parameters of glycolysis based on the targets of each successive drug injection.

### 4.8. Steady-State ATP Analysis

Steady-state intracellular ATP concentration was determined by firefly luciferin bioluminescence using the ATP Determination Kit (Invitrogen) as previously described [21].

### 4.9. Intracellular Reactive Oxygen Species (ROS) Levels

Intracellular ROS levels were determined using the Fluorometric Intracellular ROS Kit (MAK145-1KT, Sigma). Then 5 × 10^5^ cells were harvested per cell line at 500× *g* for 5 min, resuspended in 360 µL PBS, and 90 µL loaded into triplicate wells on a 96 well black, clear flat bottom plate. One hundred microliters of reaction mix prepared according to manufacturer’s instructions was added to each well, the plate covered from light and incubated for 1 h at 37 °C with 5% CO_2_. The fluorescence was then read at excitation 520 nm emission 605 nm in the Clariostar microplate reader as a measure of intracellular ROS. C105 was arbitrarily included as an internal normalization control for between-experiment variation. The fluorescence is proportional to the amount of ROS present.

### 4.10. Mitochondrial or EBV Genome Copy Number

Relative mitochondrial genome copy number was determined by the amplification and detection of two indicative mitochondrially encoded genes (*mtND1* and *mtND4*) as previously described [21].

Relative EBV genome copy number was determined by the amplification and detection of two indicative EBV genes (*BHRF* and *EBNA-1)* [65] following the same qPCR method as previously described [21].

### 4.11. 4E-BP1 Phosphorylation Levels (TORC1 Activity)

TORC1 activity in ME/CFS lymphoblast lysates was measured using a Time-resolved FRET-based multiwell plate assay of the phosphorylation state of 4E-BP1, a major TORC-1 substrate (Cisbio Bioassays). Cells were harvested, resuspended in growth medium at 2.75 × 10^5^ cells/mL and plated in four replicates at 5 × 10^4^ cells/well in a 96-well plate. Cells were incubated at 5% CO_2_/37 °C for 2 h, with two of the replicates subjected to TOR inhibition by 0.5 µM TORIN2. Lysis buffer was added to each well as per manufacturer instructions and the plate mixed on an orbital shaker for 40 min before plating each sample into a 384 well white plate (Corning)—incorporating various controls and antibody mix (anti- 4E-BP1 antibody labelled with d2 acceptor, and anti-phospho-4E-BP1 antibody labelled with Eu^3+^-cryptate donor) according to manufacturer instructions. After a 2 h incubation at room temperature the plate was scanned by the Clariostar plate reader (BMG) and the ratio of the FRET signal from anti-phospho-4E-BP1 antibody to the donor fluorescence signal from anti-4E-BP1 antibody was measured according to instructions. C105 cells were included as an internal normalization control for between-experiment variation.

### 4.12. Western Blotting

Cells were lysed in SDS loading buffer (63 mM Tris hydrochloride, 10% glycerol, 2% SDS, 10% mercaptoethanol and 0.0001% Bromophenol Blue) with a protease inhibitor cocktail (complete EDTA-free, Roche). A small aliquot of each sample was briefly sonicated and analyzed for total protein concentration using a Qubit Protein Assay Kit and Qubit 2.0 Fluorometer (Thermo-Fisher Scientific) according to the manufacturer’s instructions.

The samples were then heated to 90 °C for 10 min and 30 µg of total protein was loaded into each well in 12% SDS polyacrylamide gels. After electrophoresis, proteins were transferred onto PVDF membranes (Amersham Hybond-P, GE Healthcare) using a Trans Blot Turbo Blotting apparatus (Bio-Rad) for 30 min at 180 V, 1.0 A, blocked for 1h with blocking buffer (5% skim milk, TBS) and incubated overnight with primary antibodies (Total OXPHOS human WB antibody cocktail, Abcam, ab110411) diluted 1:1000 in blocking buffer. This cocktail is directed against five OXPHOS proteins. Stain-free gel scans were utilized as the internal loading control in combination with an Alexa Fluor 800-labelled secondary antibody for detection (Alexa Fluor 800 goat anti-mouse IgG diluted 1:1000 in TBS) (Thermo-Fisher Scientific). Following incubation with antibodies, the membranes were washed three times with TBS buffer containing 0.5% Tween 20, scanned with a ChemiDoc (Bio-Rad) and analyzed using the Image Lab software (Bio-Rad). Two arbitrarily selected control cell lines (C105 and C0002, two of the study controls) were included in every blot as internal normalization controls for between-experiment variation.

### 4.13. Whole Cell Proteomics

Each sample (3 × 10^6^ cells in 100 µL PBS) was dried using a SpeedVac Concentrator and Savant Refrigerated Vapor trap (Thermo-Fisher Scientific). Samples were resuspended in 8 M Urea, 100 mM Tris pH = 8.3. 1 µL of TCEP (tris [2-carboxyethyl] phosphine hydrochloride, 200 mM solution in water) was then added to the samples and incubated overnight at 21 °C in a ThermoMixer (Eppendorf AG). Four microliters of 1 M IAA (iodoacetamide in water) was added the following day and incubated in the dark at 21 °C. Next, 500 µL of 50 mM Tris (pH 8.3) and 1 μg trypsin was added to samples and left for 6 h at 37 °C in an incubator. Another 1 μg trypsin was added for double digestion and incubated overnight at 37 °C. The digested samples were purified for mass spectrometry analysis prior to peptide reconstitution and separation using Sep-Pak light C18 cartridges (Waters) according to manufacturer standard procedures. Data were collected on a Q Exactive HF (Thermo-Fisher Scientific) in Data Dependent Acquisition mode using m/z 350–1500 as MS scan range at 60,000 resolution, HCD MS/MS spectra were collected for the 15 most intense ions per MS scan at 15,000 resolution with a normalized collision energy of 28% and an isolation window of 1.4 m/z. Dynamic exclusion parameters were set as follows: exclude isotope on, duration 30 s and peptide match preferred. Other instrument parameters for the Orbitrap were MS maximum injection time 30 ms with AGC target 3 × 10^6^, for a maximum injection time of 25 ms with AGT target of 1 × 10^5^. Raw files consisting of high-resolution MS/MS spectra were processed with MaxQuant version 1.6.1.0 to detect features and identify proteins using the search engine Andromeda. UniProtKB/Swiss-Prot Homo sapiens sequence data was used as the database for the search engine. To assess the false discovery rate (FDR) a decoy data set was generated by MaxQuant after reversing the sequence database. Theoretical spectra were generated using the enzyme as trypsin allowing two missed cleavages. The minimum required peptide length used was seven amino acids. Carbamidomethylation of Cys was set as a fixed modification, while *n*-acetylation of proteins and oxidation of Met were set as variable modifications. Precursor mass tolerance was set to 5 ppm and MS/MS tolerance to 0.05 Da. The “match between runs” option was enabled in MaxQuant to transfer identifications made between runs on the basis of matching precursors with high mass accuracy. PSM and protein identifications were filtered using a target-decoy approach at a false discovery rate (FDR) of 1%.

### 4.14. Quantification and Statistical Analysis

Data was analyzed using Microsoft Excel with the Winstat add-in (http://www.winstat.com) or R using the packages R Commander [66], REzy [67] and stats. Proteomics data was analyzed employing the software Scaffold (Proteome Software) and detected proteins were identified as belonging to a single functional group (e.g., TCA cycle) or respiratory complex using the NCBI GO annotation database [68]. Unless otherwise specified, two-sample tests used the Welch *t*-test. ANOVA and Fisher’s exact tests were used as specified and appropriate. The significance of individual coefficients in multiple regression analysis was tested using *t*-tests. The binomial test was employed to assess whether all detected proteins in a single functional group or respiratory complex were coordinately up- or down-regulated in the ME/CFS group compared to controls, the null hypothesis being that the levels of each protein had a probability of 0.5 of being above the control average. The single sample *t* test was also used to assess whether the average fold change in the levels of all detected proteins in a single functional group or respiratory complex in the ME/CFS cohort was significantly greater than the control value of 1.0.

## 5. Conclusions

Our results show that in ME/CFS lymphoblasts, there is an isolated Complex V inefficiency in ATP synthesis at the final step in mitochondrial oxidative phosphorylation. This is accompanied by multiple homeostatic compensations, including increased respiratory capacity, Complex V expression and capacity for fatty acid β oxidation. Together, these compensatory changes appear to be sufficient to meet the normal needs of active metabolism despite the inefficiency of ATP synthesis by Complex V. Thus, the steady state ATP levels and absolute ATP synthesis rates were both close to normal in these ME/CFS cells. However, this may leave the cells less able to respond to further acute increases in ATP demand, because the signaling and metabolic pathways involved are already chronically upregulated. AMPK activity in muscle cells cultured from CFS patients (Fukuda criteria) is reportedly unresponsive to electrical pulse-induced contraction in vitro, but not because AMPK itself is unresponsive to activation by either a mitochondrial Complex I inhibitor (metformin) or a direct AMPK activator (compound 991) [62,69]. The authors suggested that the unresponsiveness of CFS cells to additional energy demands thus seems to lie elsewhere. One possibility is the already elevated TORC1 activity, since TORC1 is an inhibitor of upstream pathways that activate AMPK. In any case, if this “cellular chronic fatigue” is present in other cell types, it may contribute to the unexplained fatigue experienced by ME/CFS patients, as suggested by the fact that all of the mitochondrial abnormalities we observed were correlated with the severity of patient symptoms measured by the Weighted Standing Time. These correlations also verify that the mitochondrial abnormalities we have found are of clinical relevance to the underlying cytopathological mechanisms and can serve as biomarkers of disease.

## Supplementary Materials

Supplementary materials can be found at https://www.mdpi.com/1422-0067/21/3/1074/s1.
