# Supplementary material for: An Isolated Complex V Inefficiency and Dysregulated Mitochondrial Function in Immortalized Lymphocytes from ME/CFS Patients
Source: Int J Mol Sci. 2020 Feb 6;21(3):1074. doi: 10.3390/ijms21031074 (PMC7036826; doi:10.3390/ijms21031074)
Supplement: Supplementary file 1 [file ijms-21-01074-s001.pdf]

Supplemental Data

Table S1: Expression of OXPHOS complex subunits in whole cell proteomes from ME/CFS patients compared to healthy controls. Binomial test of fraction upregulated with H_0 set to 0.5. Fold-change data from Scaffold using normalized iBAQ results - single sample *t* test with H_0 as mean fold change ≤ 1 and H_1 as mean fold change >1 (subunits detected in fewer than 5 samples not included).
*In any sample – some subunits were detected in only a few samples.

	**Number of subunits**	**Number of subunits detected***	**Fraction upregulated in ME/CFS proteomes**	**Binomial test *p***	**Mean fold change (± standard error)**	**Single sample *t* test *p***
Complex I	44	31	23/31	0.01	1.56±0.18	2.5×10^{-3} (n=26)
Complex II	4	3	3/3	0.25	1.2±0	NA
Complex III	10	7	5/7	0.45	1.11±0.10	0.17 (n=7)
Complex IV	19	15	7/15	1.0	1.08±0.07	0.13 (n=10)
Complex V	20	12	11/12	6.3×10^{-3}	1.13±0.09	3.0×10^{-3} (n=9)

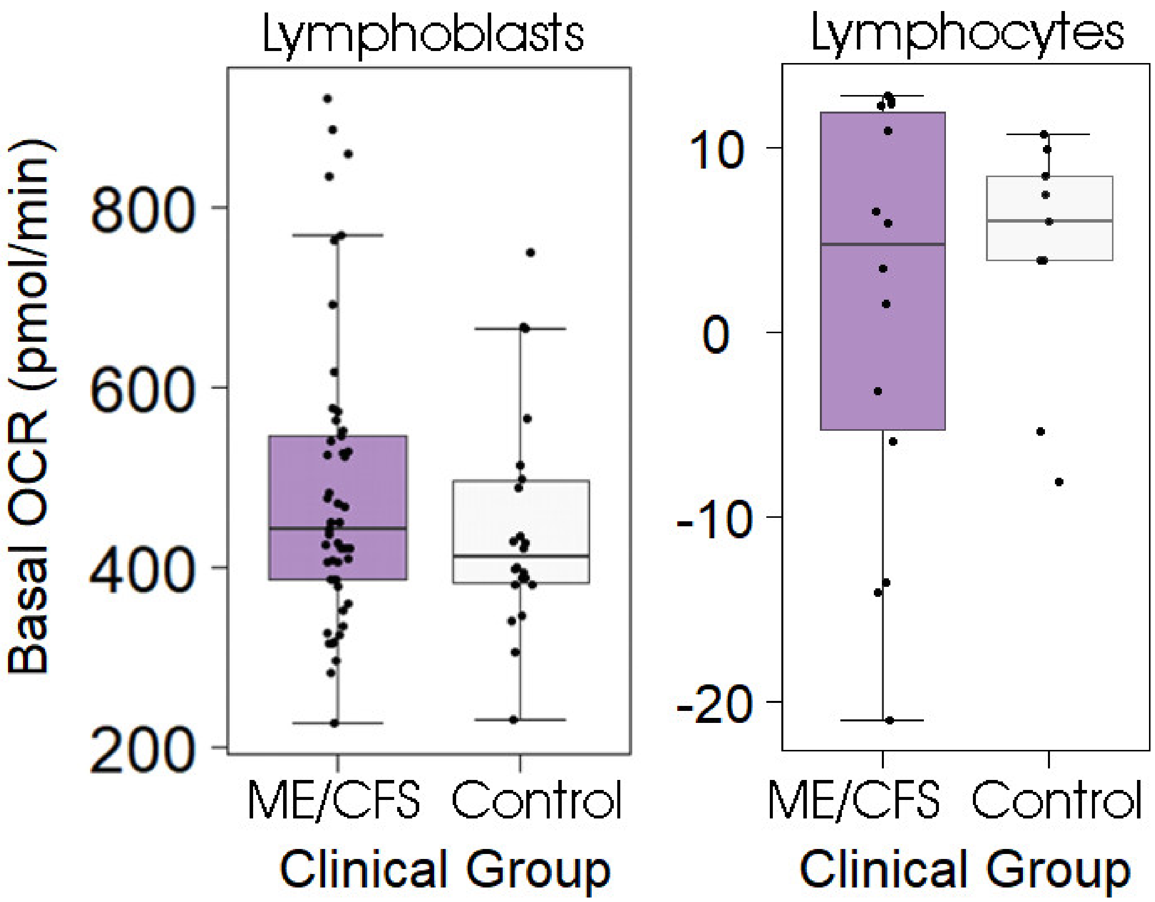

Figure S1. Box and jitter dot plots corresponding to Figure 1, showing the basal OCR of lymphoblasts and lymphocytes. The boxes show the median and the 25th and 75th percentiles, so that

the height of the box is the interquartile range (IQR). The whiskers extend to the most extreme observations (largest and smallest) falling within ±1.5xIQR of the box. Other details are described in the corresponding Figures. Each dot represents either an individual lymphoblast cell line or the lymphocytes isolated from one participant.

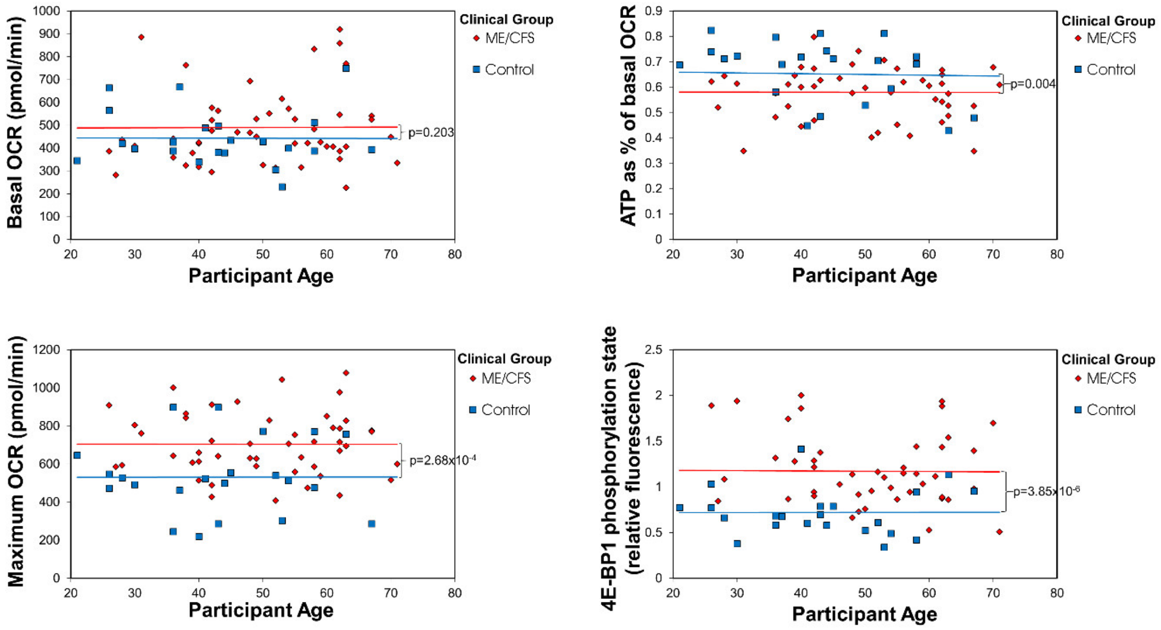

Figure S2: Participant age had no effect on key parameters of mitochondrial function and TORC1 signalling in either ME/CFS or control lymphoblasts.

Multiple regression analysis was performed with dummy variables allowing both slopes and intercepts to differ between groups, with successive removal of least significant regression variables until only significant coefficients remained. Each point represents the mean result of at least three independent experiments using lymphoblasts from a single participant. Except for the basal O_2 consumption rate (OCR), the y-intercepts differed significantly between the groups (significance probabilities shown in each panel). The slopes of the linear regressions were not significantly different from one another or from 0 for either the ME/CFS or control group ($p>0.05$).

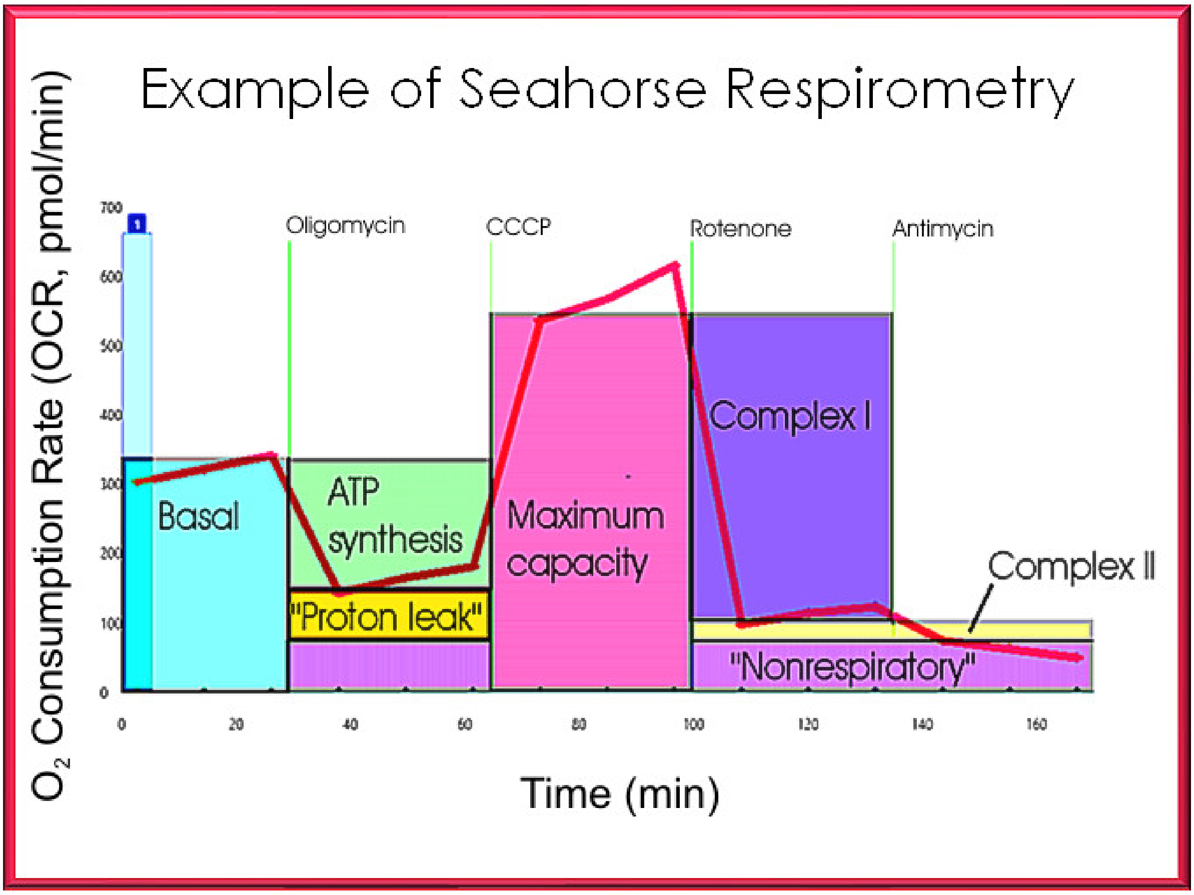

Figure S3. Example of Seahorse respirometry. Lymphoblasts ($8x10^5$ viable cells) were seeded into each of 4 wells in the 24 well plate as described previously [19]. Four wells without cells were used to measure the background signal which was subtracted from the average signal from the 4 test wells. At each assay time point the medium and drug additions were mixed (3 min), cells rested (2 min) and O_2 consumption rates measured (3 min). The 4 sequential drug additions occurred at the indicated times – oligomycin, CCCP, rotenone and antimycin A. The various respiratory parameters shown were calculated as illustrated from the measured OCRs and the changes in the OCR after each drug addition.

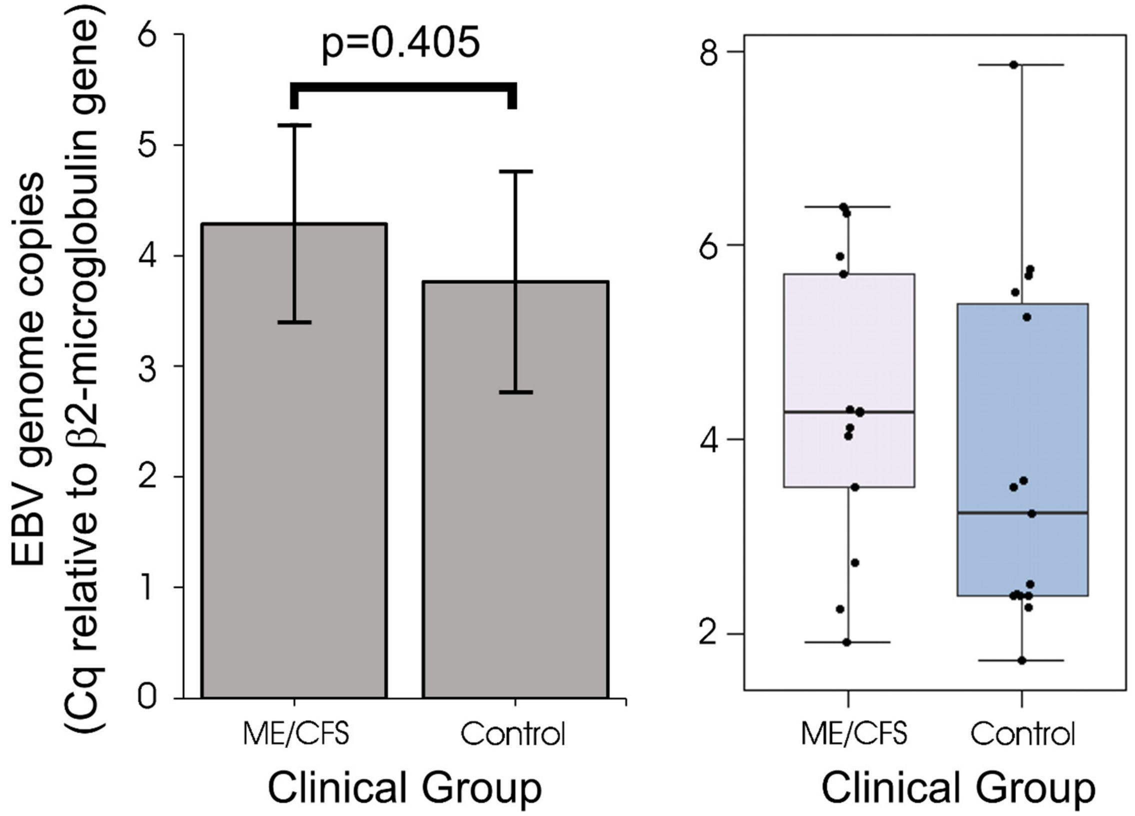

Figure S4: The genome copy number of EBV gene *BHRF-1* is unchanged between ME/CFS and control lymphoblasts. Genome copy numbers (qPCR for the EBV gene *BHRF-1* relative to nuclear -microglobulin gene) are unchanged in ME/CFS lymphoblasts (independent *t*-test, $p>0.05$ in both cases). Each ME/CFS (n=13) and control cell line (n=15) was assayed by qPCR in multiple independent experiments. Error bars represent 95% confidence intervals. Similar results were obtained in qPCR experiments measuring the relative copy numbers for the *EBNA-1* gene (not shown). The boxes show the median and the 25th and 75th percentiles, so that the height of the box is the interquartile range (IQR). The whiskers extend to the most extreme observations (largest and smallest) falling within $\pm$1.5xIQR of the box. Other details are described in the corresponding Figures. Each dot represents an individual lymphoblast cell line.

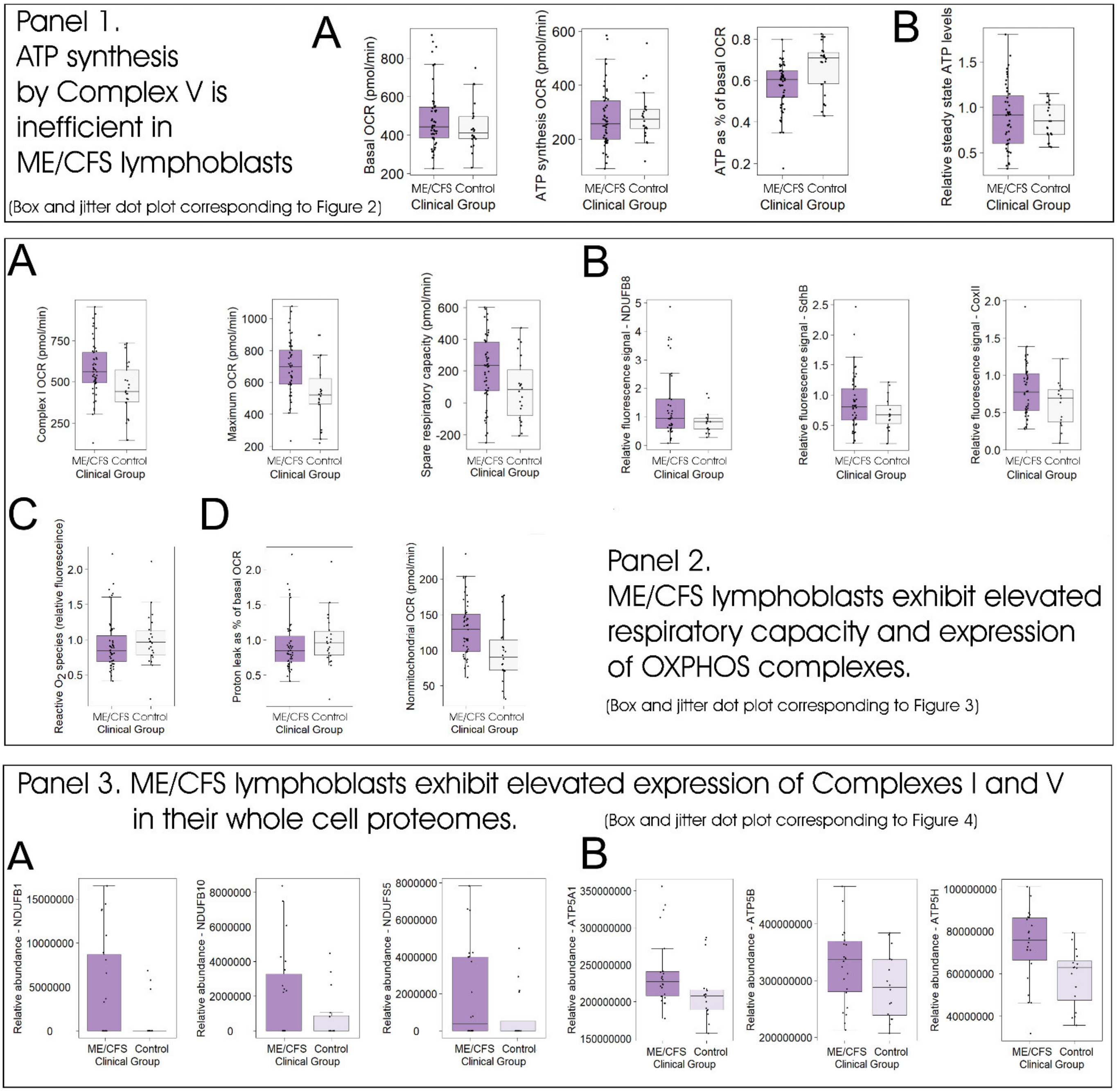

Figure S5. Box and jitter dot plots corresponding to Figures 2, 3 and 4A,B. The boxes show the median and the 25th and 75th percentiles, so that the height of the box is the interquartile range (IQR). The whiskers extend to the most extreme observations (largest and smallest) falling within ±1.5xIQR of the box. Other details are described in the corresponding Figures. Each dot represents an individual lymphoblast cell line.

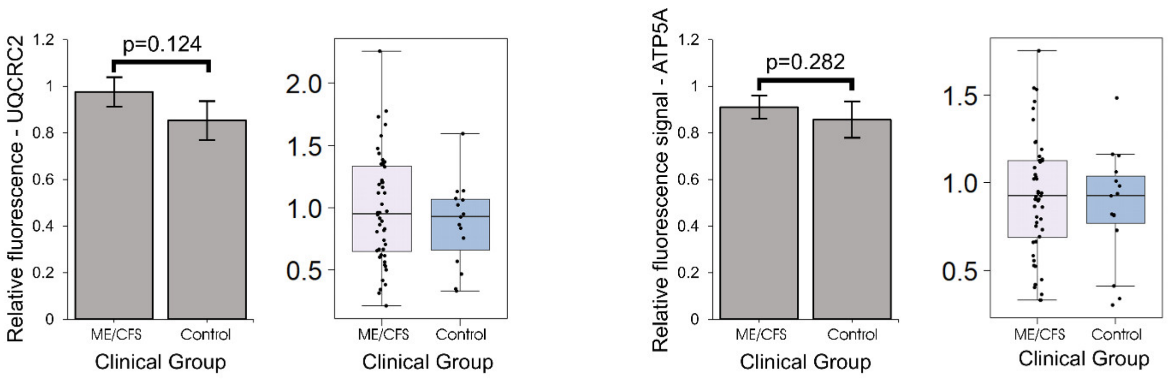

Figure S6: Complex III subunit UQCRC2 and Complex V subunit ATP5A expression was not significantly elevated in semiquantitative western blots (independent t-test). Each ME/CFS (n=48) and control (n=17) cell line was assayed in at least three independent experiments and means ±SEM were calculated (bar graphs). Western blot data is expressed in relative terms as each experiment was normalised to internal loading controls. Corresponding box and jitter dot plots are shown to the right of each bar graph. The boxes show the median and the 25th and 75th percentiles, so that the height of the box is the interquartile range (IQR). The whiskers extend to the most extreme observations (largest and smallest) falling within ±1.5xIQR of the box. Other details are described in the corresponding Figures. Each dot represents an individual lymphoblast cell line.

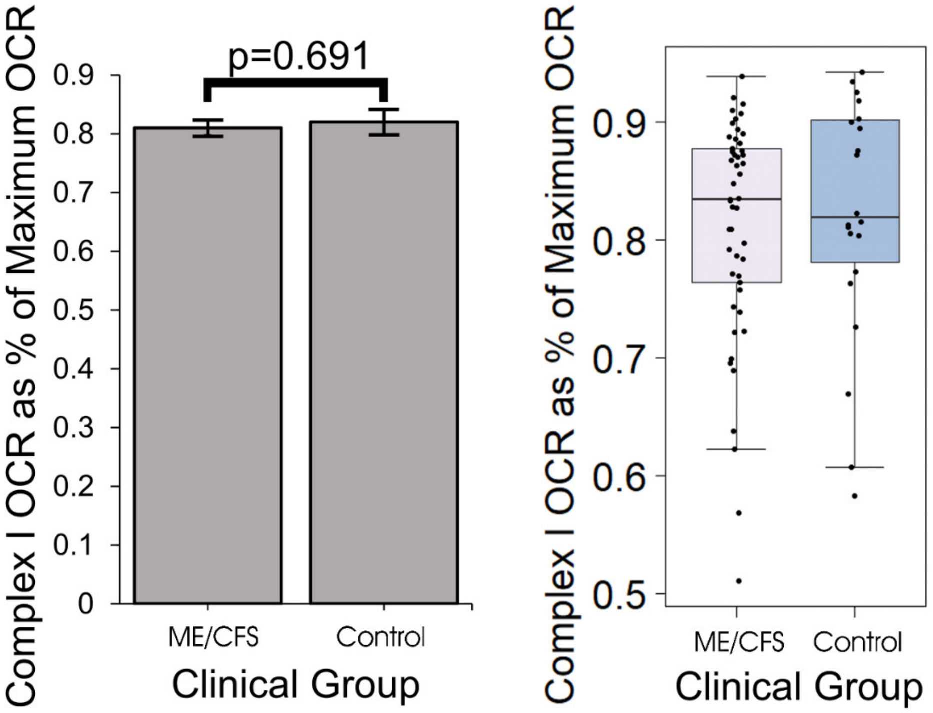

Figure S7: Complex I OCR as a percentage of the uncoupled maximum OCR was unchanged in ME/CFS lymphoblasts (independent t-test).

The OCR was measured in lymphoblasts from ME/CFS and control individuals by the Seahorse XFe24 Extracellular Flux Analyser. Each ME/CFS (n=50) and control (n=22) cell line was assayed over four replicates in at least three independent experiments and means ±SEM were calculated (bar graphs). The corresponding box and jitter dot plots are shown in the right panel. The boxes show the median and the 25th and 75th percentiles, so that the height of the box is the interquartile range (IQR).

The whiskers extend to the most extreme observations (largest and smallest) falling within 1±1.5xIQR of the box. Other details are described in the corresponding Figures. Each dot represents an individual lymphoblast cell line.

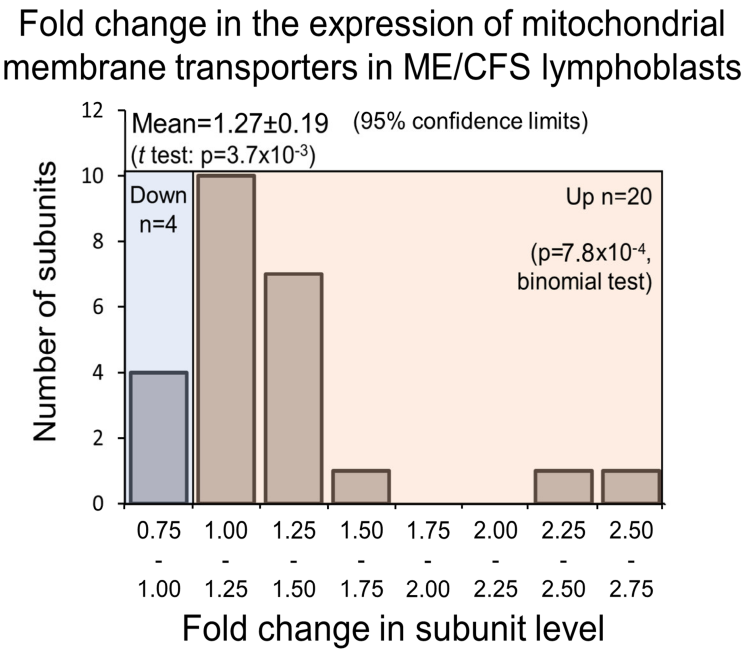

Figure S8: Elevated expression of mitochondrial membrane transporters in whole cell ME/CFS proteomes. All detected members of the SLC25 mitochondrial transporter protein family were included, as were all detected proteins whose GO annotation matched the terms "mitochondrion", "mitochondrial membrane part" and "mitochondrial transport". The resulting list included 10 SLC25 transporters and 8 subunits of the mitochondrial protein import complexes TIMM, TOMM and SAMM. Respiratory complex subunits were removed from the list as were non-integral membrane proteins not directly involved in transport. Fold-change data from Scaffold using normalized iBAQ results. The fraction of detected mitochondrial transport proteins that were upregulated (binomial test with H_o set to 0.5) and the average extent of the upregulation (single sample *t* test with H_o $m \leq 1$ and H_1 $m>1$) were significant at the indicated levels.

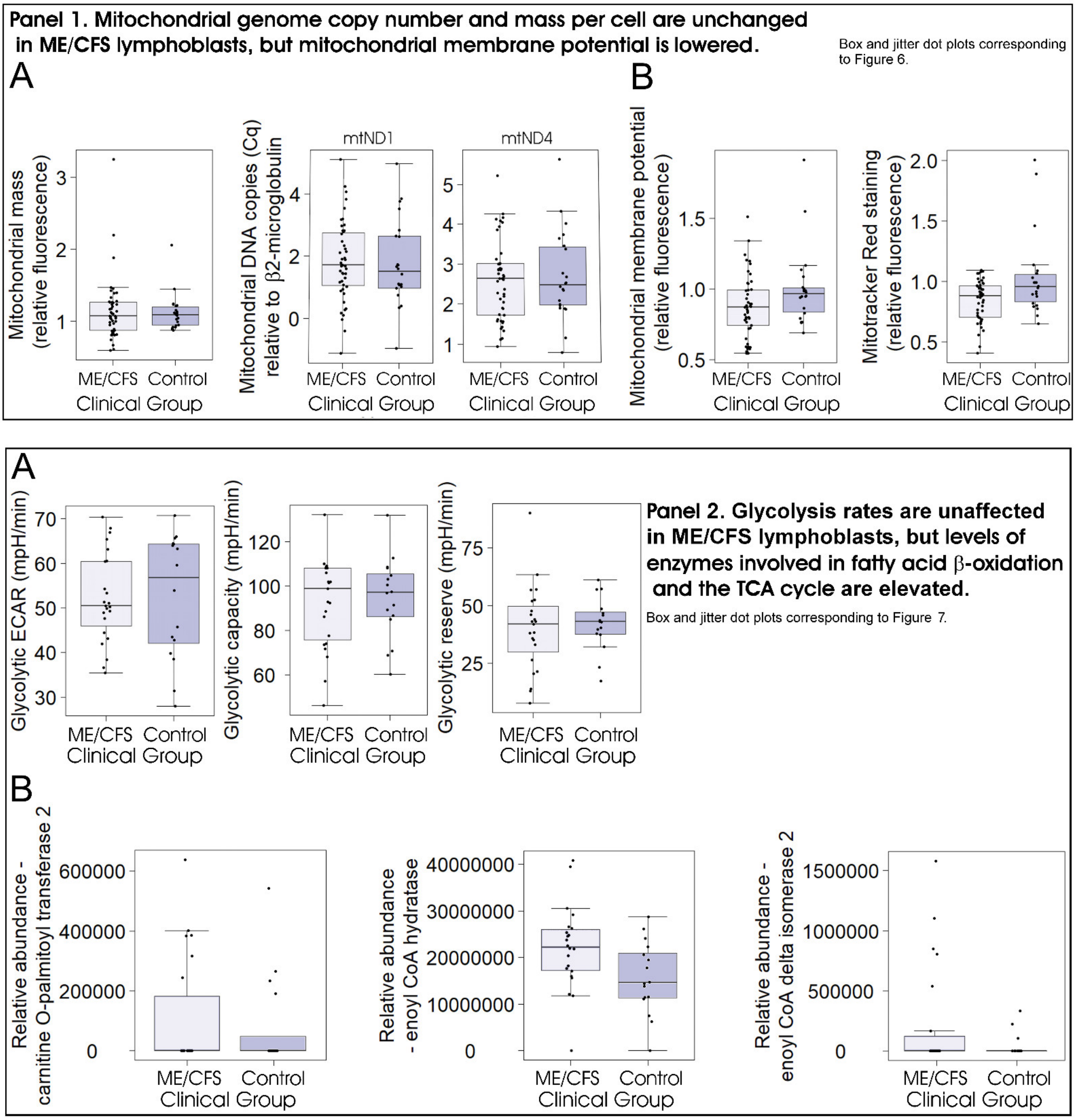

Figure S9. Box and jitter dot plots corresponding to Figures 6 and 7A,B. The boxes show the median and the 25th and 75th percentiles, so that the height of the box is the interquartile range (IQR). The whiskers extend to the most extreme observations (largest and smallest) falling within $\pm$1.5xIQR of the box. Other details are described in the corresponding Figures. Each dot represents an individual lymphoblast cell line.

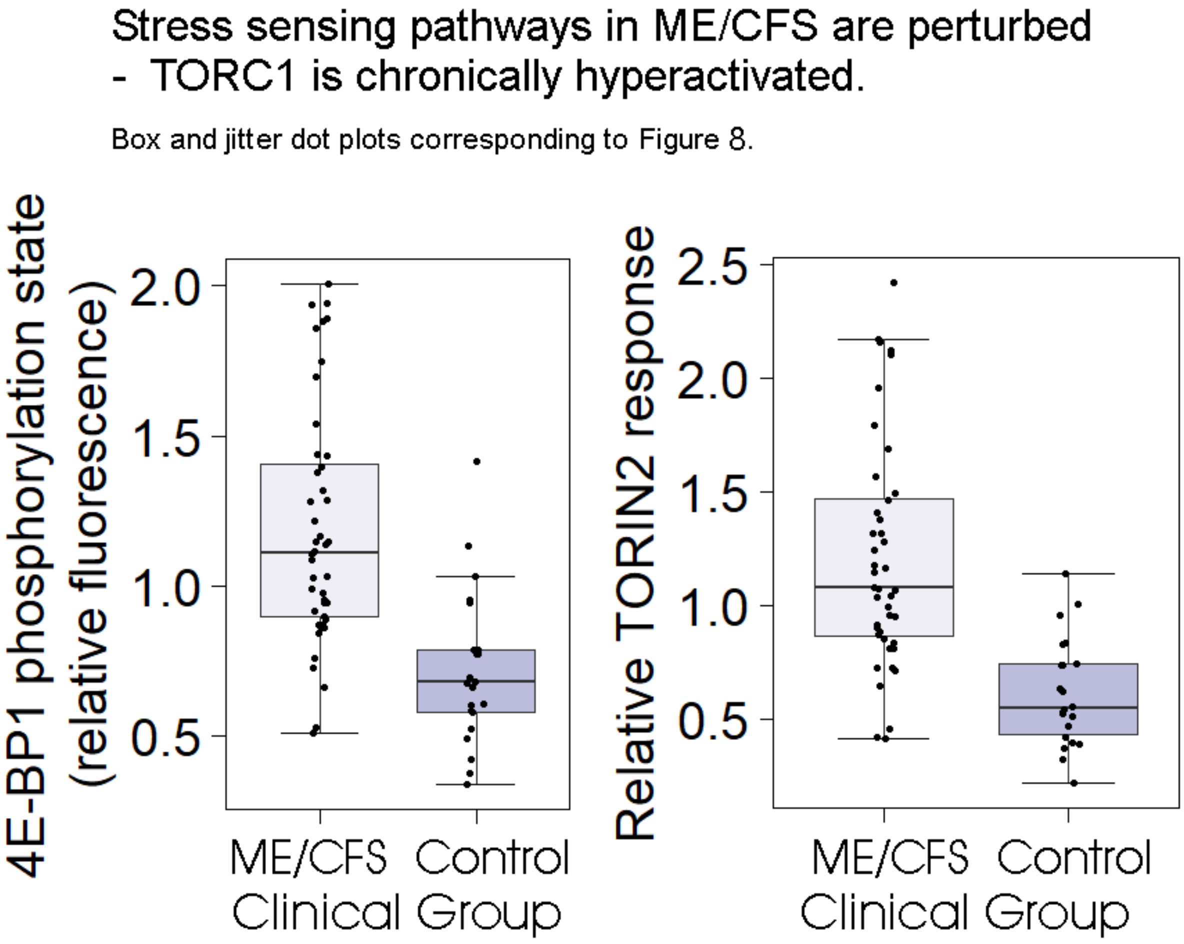

Figure S10. Box and jitter dot plots corresponding to Figure 8. The boxes show the median and the 25th and 75th percentiles, so that the height of the box is the interquartile range (IQR). The whiskers extend to the most extreme observations (largest and smallest) falling within ±1.5xIQR of the box. Other details are described in the corresponding Figures. Each dot represents an individual lymphoblast cell line.
